# CCT3 acts upstream of YAP and TFCP2 as a potential target and tumour biomarker in liver cancer

**DOI:** 10.1038/s41419-019-1894-5

**Published:** 2019-09-09

**Authors:** Ya Liu, Xiao Zhang, Jiafei Lin, Yuxin Chen, Yongxia Qiao, Susu Guo, Yueyue Yang, Guoqing Zhu, Qiuhui Pan, Jiayi Wang, Fenyong Sun

**Affiliations:** 1 Department of Clinical Laboratory, Shanghai Tenth People’s Hospital of Tongji University, 200072 Shanghai, China; 2 Shanghai Institute of Thoracic Tumours, Shanghai Chest Hospital, Shanghai Jiaotong University, 200030 Shanghai, China; 3 Department of Clinical Laboratory, Shanghai Ruijin Hospital, Shanghai Jiaotong University, 200025 Shanghai, China; 4 School of Public Health, Shanghai Jiaotong University School of Medicine, 200025 Shanghai, China; 5 Department of Laboratory Medicine, Shanghai Children’s Medical Center, Shanghai Jiaotong University School of Medicine, 200127 Shanghai, China

**Keywords:** Tumour biomarkers, Transcriptional regulatory elements

## Abstract

Although Yes-associated protein (YAP) is very important to liver cancer, its nuclear localisation prevents consideration as a promising therapeutic target and a diagnostic biomarker. Recently, we reported that the protumourigenic roles of YAP in liver cancer are indispensable for transcription factor CP2 (TFCP2) in a Hippo-independent manner; however, proteins that act upstream to simultaneously control YAP and TFCP2 remain unclear. The aim of this study was to uncover such proteins and evaluate whether they are potential YAP-associated therapeutic targets and diagnostic biomarkers. Mass spectrometry revealed that chaperonin containing TCP1 subunit 3 (CCT3) co-interact with YAP and TFCP2, and notably, CCT3 is a non-nuclear protein. CCT3 was elevated in liver cancer, and its higher expression was associated with poorer overall survival. Inhibiting CCT3 resulted in a suppressed transformative phenotype in liver cancer cells, suggesting that CCT3 might be a potential therapeutic target. CCT3 prolonged half-life of YAP and TFCP2 by blocking their ubiquitination caused by poly(rC) binding protein 2 (PCBP2) in a beta-transducin repeat containing E3 ubiquitin protein ligase (βTrCP)-independent manner. Interestingly, PCBP2 directly interacted with YAP via a WB motif-WW domain interaction, whereas indirectly interacted with TFCP2 via the aid of YAP. Furthermore, CCT3 was capable of separating PCBP2-YAP interactions, thereby preventing YAP and TFCP2 from PCBP2-induced ubiquitination. Moreover, YAP and TFCP2 were downstream of CCT3 to positively control tumourigenesis, yet such effects were inhibited by PCBP2. Clinically, CCT3 was positively correlated with YAP and TFCP2, and elevated levels of the CCT3-YAP-TFCP2 axis might be critical for liver malignancy. In addition, seral-CCT3 was proven to be a potential biomarker, and its diagnostic capacity was better than that of alpha fetoprotein (AFP) to a certain extent. Together, CCT3 acts as a trigger of YAP and TFCP2 to affect tumourigenesis and serves as a potential therapeutic target and biomarker in liver cancer.

## Introduction

Yes-associated protein (YAP) is required for liver tumourigenesis^1–3^ and the maintenance of tumour growth;^4,5^ however, YAP is negatively regulated by Hippo signalling^6,7^. The activation of Hippo leads to cytosolic retention and ubiquitination by ubiquitin E3 ligases, such as βTrCP^8^. YAP has emerged as an attractive therapeutic target to treat liver cancer. However, YAP targeted therapy is lacking, although small molecules have been sought to potentially inhibit YAP activation^9,10^. Unfortunately, such drugs still lack ideal selectivity. Additionally, no definite monoclonal antibody-based therapeutics against YAP have been developed thus far. This might be because YAP exerts its protumourigenic roles mostly in the nucleus^11,12^, and nuclear YAP cannot be efficiently captured by monoclonal antibodies. In contrast, proteins on the cell surface or in the cytoplasm are better targets for therapeutic antibodies^13,14^. YAP is also not a serum biomarker because it cannot be detected in the serum of liver cancer patients^15^. This effect might also be because YAP is a nuclear protein and cannot be easily released into the blood flow. Although the YAP-associated cell membrane protein CD166 and the melanoma cell adhesion molecule (MCAM) have been identified as serum biomarkers for liver cancer^15–17^, traditional AFP cannot be easily replaced^18,19^.

Recently, TFCP2 has been identified to act as a YAP co-factor to stimulate YAP-dependent liver malignancy^20^. TFCP2 facilitates the transcription of YAP downstream proto-oncogenes^20^. However, the upstream proteins, especially those belonging to the cytosol and cell membrane compartment, which co-regulate YAP and TFCP2 are still unknown. Identifying such proteins might provide new ways to treat YAP-associated liver cancer and highlight potential biomarkers to increase the sensitivity of AFP, CD166 and MCAM to diagnose liver cancer.

Here, CCT3 was identified to act as an upstream trigger of YAP and TFCP2. CCT3 is a potential therapeutic target, and seral-CCT3 might be a promising biomarker for liver cancer screening and diagnosis.

## Results

### CCT3 interacted with both YAP and TFCP2 and was upregulated in liver cancer and correlated with poor patient survival

**Fig. 1:**
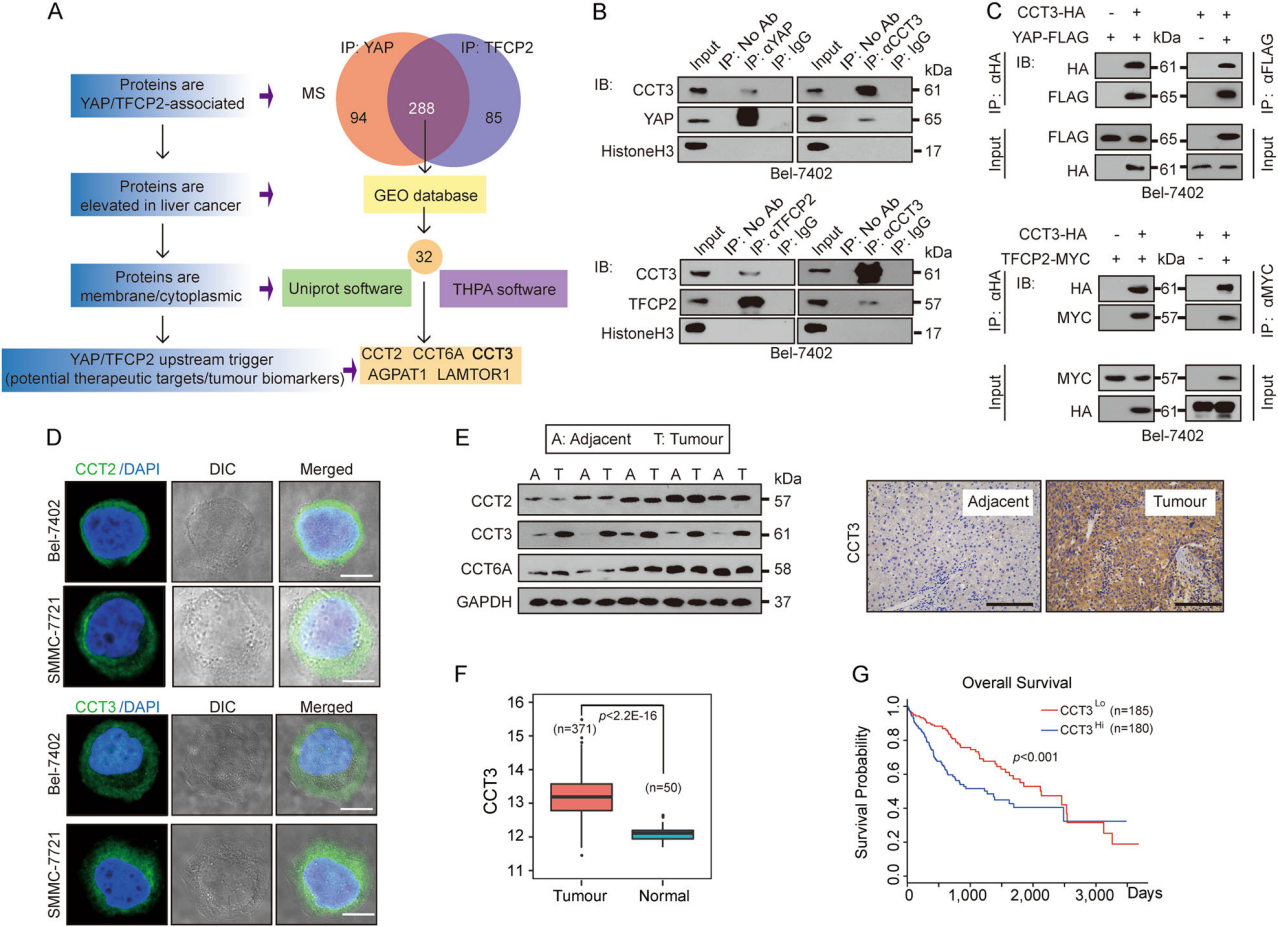
CCT3 interacted with YAP and TFCP2 and was critical for liver cancer. **a** MS and bioinformatics revealed that CCT3 was associated with both YAP and TFCP2 in Bel-7402 cells, and might located on the membrane or in the cytoplasm. **b** Endogenous CCT3 interacted with endogenous YAP and TFCP2, as measured by co-IP in Bel-7402 cells. **c** Exogenous CCT3-HA interacted with YAP-FLAG and TFCP2-MYC, as measured by reciprocal co-IP in Bel-7402 cells. **d** The sub-cellular localisation of CCT2 and CCT3 in Bel-7402 and SMMC-7721 cells. Scale bar, 20 μm. **e** CCT3 was highly upregulated in liver cancer compared to adjacent liver, as evaluated in five paired adjacent (A) and tumourous (T) liver tissue. The representative IHC images of CCT3 are also shown on the right side. Scale bar, 200 μm. **f** The TCGA liver cancer cohort (50 normal liver, 371 liver cancer samples) identified CCT3 was upregulated in liver cancer. **g** Overall survival plots of liver cancer patients (*n* = 365, *p* < 0.001) in TCGA stratified by CCT3 expression. Images of IF, IHC and WB are representative of three independent experiments

To identify YAP and TFCP2 upstream that might be potential therapeutic targets and tumour biomarkers, we screened proteins that meet the criteria below: (1) should be YAP and TFCP2-associated; (2) should be elevated in liver cancer; (3) should be membrane or cytoplasmic (Fig. 1a). Via Mass spectrometry (MS) of the immune precipitates that immunoprecipitated with anti-YAP and anti-TFCP2 antibodies, 288 proteins were predicted to co-interact with both YAP and TFCP2 (Fig. 1a and Supplementary Table. S1). Further screening using the GEO database revealed 32 proteins that were also elevated in liver cancer (Fig. 1a). To narrow candidates, UniProt (https://www.uniprot.org) and the human protein atlas (THPA, https://www.proteinatlas.org/search) online software, which provides information on the location and topology of the mature protein within cell, were used to find membrane or cytoplasmic proteins. Five proteins, including CCT2, CCT6A, CCT3, AGPAT1 and LAMTOR1, were finally selected (Fig. 1a). By verification, only CCT proteins (CCT2, CCT3 and CCT6A), participating in protein folding^21–25^, were found to co-interact with YAP and TFCP2 (Fig. 1b, c and Supplementary Fig. S1A–E). However, CCT2 and CCT3, but not CCT6A, had a non-nuclear subcellular localisation (Fig. 1d and Supplementary Fig. S1F) in Bel-7402 and SMMC-7721 cells, both of which are widely used for the study of liver cancer^16,26–28^. By testing clinical samples, only CCT3 was elevated in tumours compared to adjacent liver tissues (non-tumour tissue just alongside the tumour tissue) (Fig. 1e). Additionally, the cancer genome atlas (TCGA) revealed that CCT3 was significantly elevated in tumours compared to normal tissues in the liver (Fig. 1f). Kaplan–Meier plots of survival revealed that patients with higher CCT3 had a worse survival than those with lower CCT3 (Fig. 1g). Collectively, we focused on CCT3 in the following study.

### CCT3 correlated with transformative phenotypes

CCT3 is indispensable for hepatocellular carcinoma (HCC) cell proliferation^23^. To confirm this finding, we knocked down and overexpressed CCT3 (Supplementary Fig. S2A). Cell proliferation was significantly reduced when CCT3 was knocked down but was induced by CCT3 overexpression (Supplementary Fig. S2B). In contrast, CCT3 had opposite effects on caspase 3/7 activity (Supplementary Fig. S2C). Furthermore, colony formation and in vivo xenograft growth were also positively correlated with CCT3 (Supplementary Fig. S2D–F). Additionally, the effects caused by knocking down CCT3 could be reversed by overexpressing CCT3 (Supplementary Fig. S2A–F).

### CCT3 regulated YAP and TFCP2 at the protein level

**Fig. 2:**
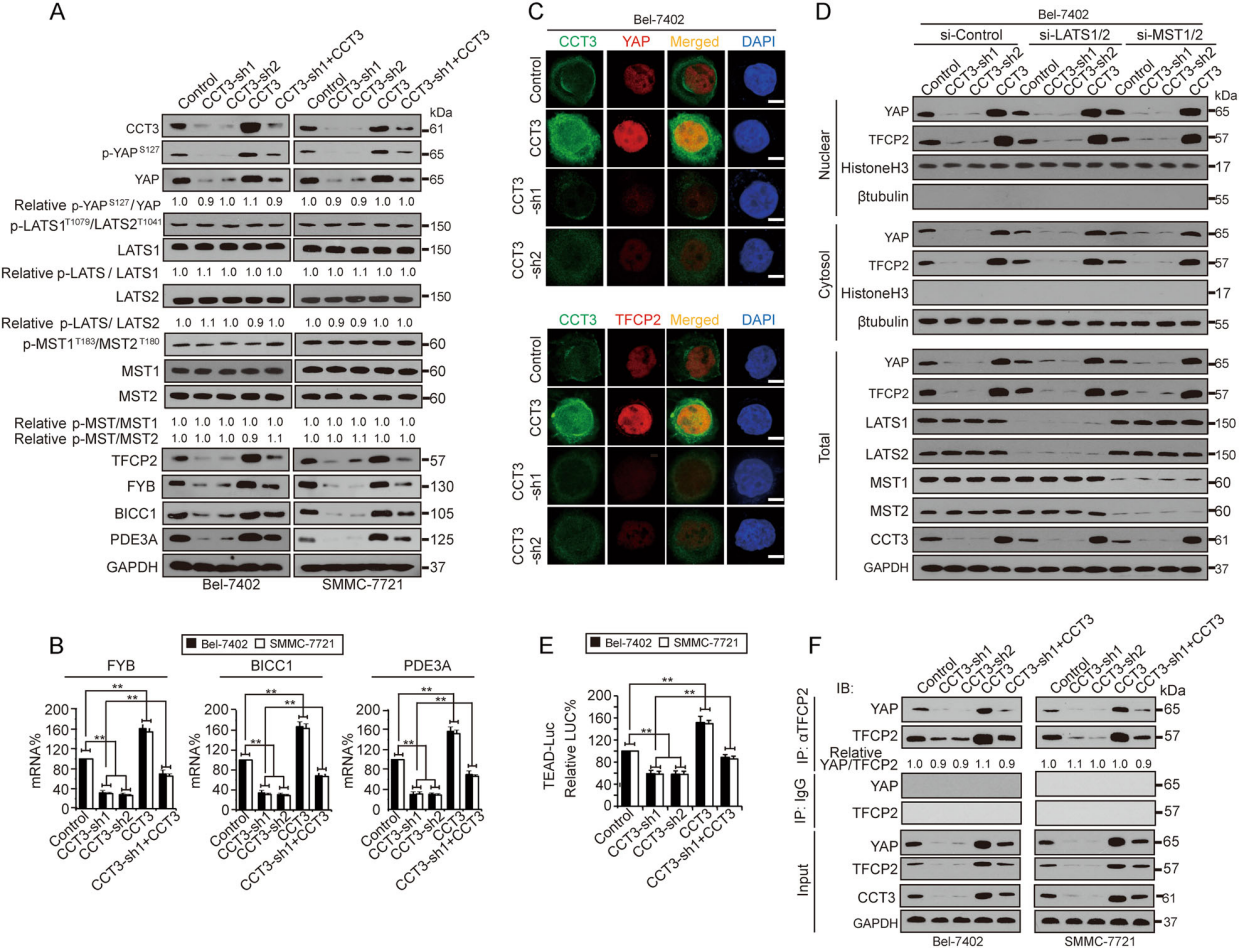
CCT3 stimulated YAP and TFCP2 independent of affecting their localisation. **a** Representative WB images of LATS, MST, YAP, TFCP2 and their co-targets of YAP and TFCP2 in control cells and Bel-7402 and SMMC-7721 cells with CCT3 knockdown or overexpression, as indicated. **b** CCT3 positively regulated targets of YAP and TFCP2. The mRNA levels of FYB, BICC1 and PDE3A were measured by qPCR in Bel-7402 and SMMC-7721 cells. **c** CCT3 regulated expression but not sub-cellular localisation of YAP and TFCP2, as measured by IF in Bel-7402 cells. Scale bar, 20 μm. **d** Nucleic-cytoplasmic fraction experiments in control cells, Bel-7402 cells with LATS1/2 and MST1/2 knockdown, as visualised by WB for YAP and TFCP2. **e** CCT3 boosted the activity of YAP, as measured indirectly by the pUAS-LUC/TEAD-Gal4 reporter system. **f** CCT3 was unable to affect the YAP-TFCP2 interaction, as measured by co-IP. Images of IF and WB are representative of three independent experiments. **p < 0.01 indicate statistical significance. The data were analysed by a one-way ANOVA test from three independent experiments

Next, we investigated whether CCT3 regulates YAP and TFCP2. YAP and TFCP2 could be suppressed by knocking down CCT3, which was rescued by CCT3 overexpression (Fig. 2a). Both the protein and mRNA of FYB, BICC1 and PDE3A, which are common targets of YAP and TFCP2^20^, were also changed in the same manner as CCT3 in cells and xenografts (Fig. 2a, b and Supplementary Fig. S2G), suggesting that CCT3 regulates YAP/TFCP2 transcription activity via affecting their expression. However, CCT3 merely regulates the YAP and TFCP2 protein because the alteration in CCT3 did not affect their mRNA (Supplementary Fig. S3A). Additionally, neither TFCP2 nor YAP reversely regulated CCT3 (Supplementary Fig. S3B–C).

Hippo inactivates YAP via cytoplasmic retention of YAP from the nucleus^29^. We tested whether CCT3 affects the translocation of YAP and TFCP2. Knocking CCT3 down only reduced the strength of fluoresce representing YAP and TFCP2; however, the nuclear localisation of YAP and TFCP2 were unaffected (Fig. 2c). The nucleic-cytoplasmic fraction experiments also demonstrated that only expression but not subcellular distribution of YAP and TFCP2 were altered by CCT3 (Fig. 2d). Furthermore, the phosphorylation and total levels of YAP were changed in parallel by CCT3 in Bel-7402 and SMMC-7721 cells (Fig. 2a) and in xenografts (Supplementary Fig. S2G), suggesting that changes in phosphorylation are a result of changes in YAP expression. Moreover, knocking down LATS1/2 and MST1/2, both of which are key components of the Hippo pathway, did not reverse YAP and TFCP2 expression resulting from alterations in CCT3 in Bel-7402 cells (Fig. 2d), confirming that LATS and MST might not be involved in controlling YAP and TFCP2 by CCT3.

Although YAP activity was further revealed to be positively regulated by CCT3, as measured by a TEAD-based luciferase reporter system (Fig. 2e), CCT3 was unable to interfere with the TFCP2-YAP interaction (Fig. 2f and Supplementary Fig. 2H). Collectively, CCT3 regulates YAP, and TFCP2 might merely affect their expression at the protein level in a LATS- and MST-independent manner.

### CCT3 enhanced the protein stability of YAP and TFCP2 via suppressing ubiquitination

**Fig. 3:**
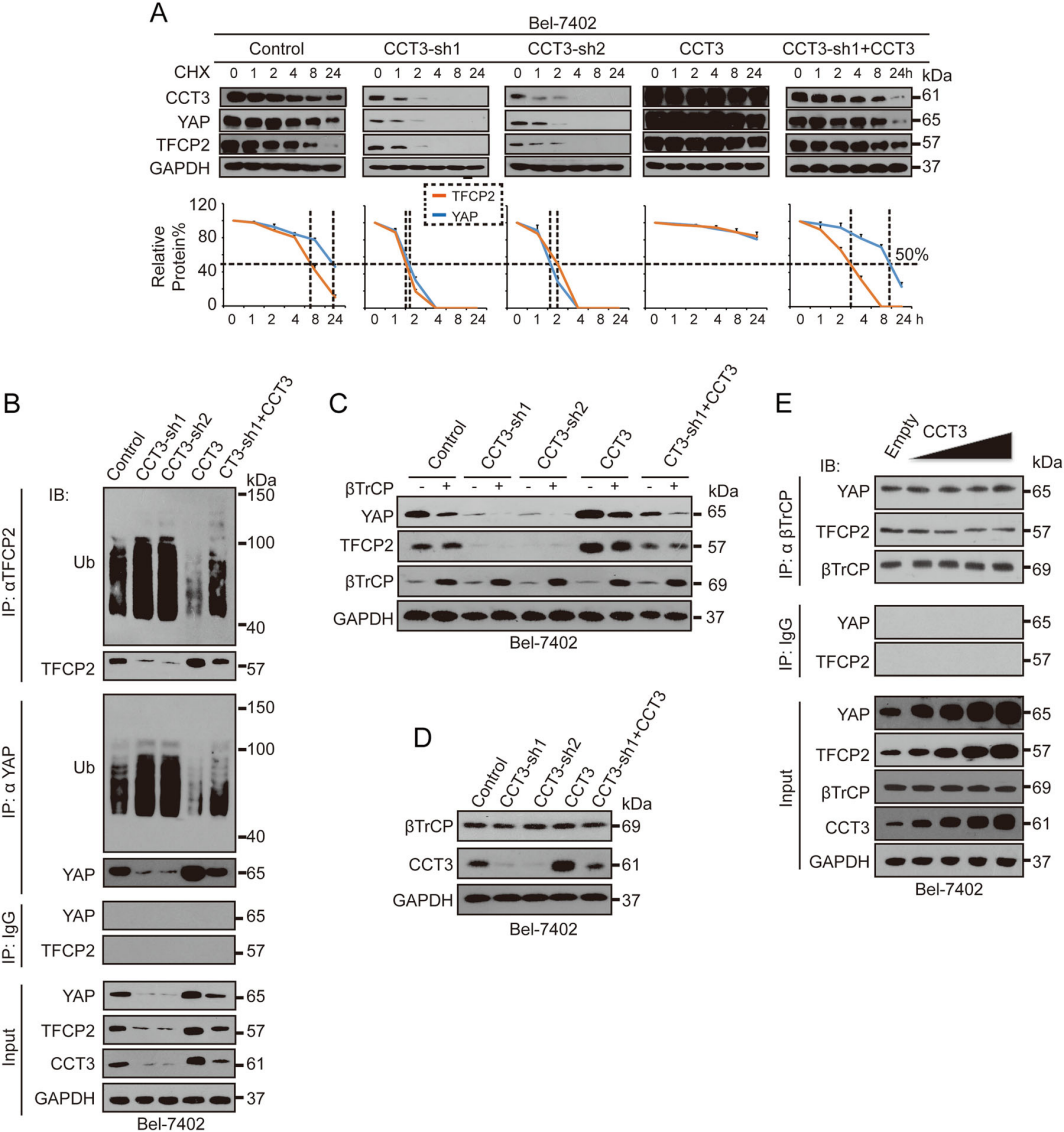
CCT3 positively regulated the protein stability of YAP and TFCP2 via ubiquitination independent of βTrCP. **a** CHX chase experiment on YAP and TFCP2 in control cells and Bel-7402 cells with CCT3 knocked down or overexpressed, as indicated. The relative protein levels were also plotted at the bottom. The relative protein levels of YAP/TFCP2 were normalised to those of GAPDH, and the “0 h” point was arbitrarily set to 100%. **b** CCT3 suppressed ubiquitination of TFCP2 and YAP, as measured by IP in Bel-7402 cells. **c** βTrCP was not involved in the CCT3-mediated regulation of YAP and TFCP2, as measured in Bel-7402 cells with or without βTrCP overexpression. **d** CCT3 did not affect βTrCP expression, as measured by WB in Bel-7402 cells. **e** CCT3 did not influence the interaction between βTrCP and YAP/TFCP2, as measured by co-IP in Bel-7402 cells. Images of WB are representative of three independent experiments

We then investigated whether CCT3 affects YAP and TFCP2 expression by influencing protein stability. Cycloheximide (CHX) chase experiments revealed that CCT3 prolonged the half-life of YAP and TFCP2 (Fig. 3a and Supplementary Fig. S4A), suggesting CCT3 affects protein stability of YAP and TFCP2. Increased protein stability usually results from decreased ubiquitination. Knocking down CCT3 increased ubiquitination of YAP and TFCP2, while overexpressing CCT3 caused the opposite outcome, and expectedly, the effects of CCT3 knockdown were rescued by CCT3 overexpression (Fig. 3b and Supplementary Fig. S4B), suggesting that CCT3 enhances the protein stability of YAP and TFCP2 might via reducing their ubiquitination.

βTrCP acts as a ubiquitin E3 ligase to ubiquitinate YAP. Does CCT3 control YAP and TFCP2 via βTrCP? Unexpectedly, βTrCP still downregulated YAP regardless of whether CCT3 was knocked down or overexpressed. Moreover, βTrCP was unable to downregulate TFCP2 (Fig. 3c and Supplementary Fig. S4C). βTrCP per se could not be regulated by CCT3 (Fig. 3d and Supplementary Fig. S4D). Additionally, CCT3 had no capacity to recruit βTrCP to YAP and TFCP2 (Fig. 3e). These results suggested that CCT3 regulates YAP and TFCP2 in a βTrCP-independent manner.

### PCBP2 was involved in CCT3 regulation of YAP and TFCP2

**Fig. 4:**
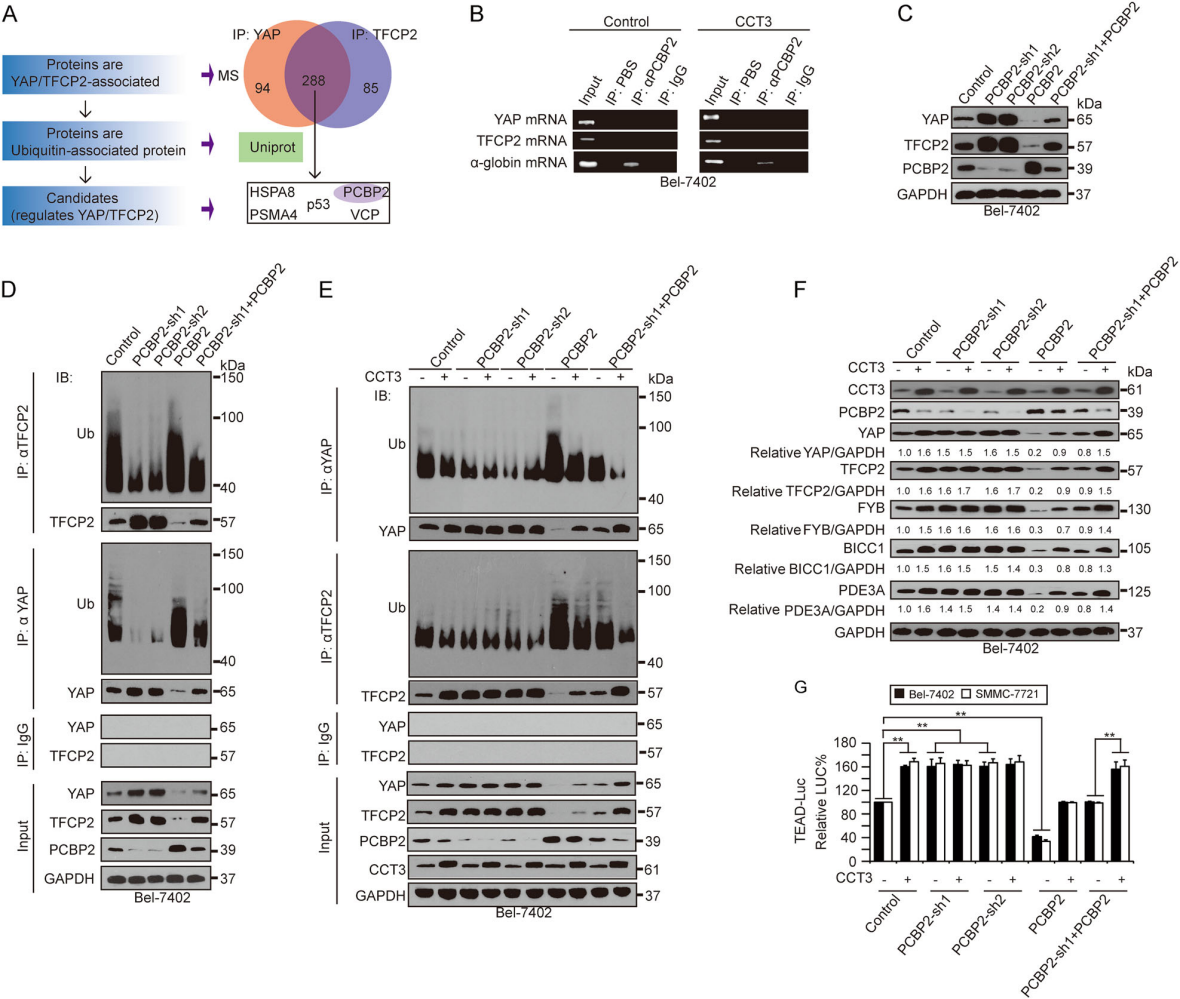
PCBP2 was involved in the regulation of YAP and TFCP2. **a** PCBP2 was identified by MS and UniProt software to be a ubiquitin-associated protein with YAP and TFCP2. **b** PCBP2 did not act as an RNA-binding protein to YAP and TFCP2, as measured by RIP in Bel-7402 cells. The α-globin mRNA was examined in parallel as a positive control. **c** PCBP2 negatively regulated the expression of YAP and TFCP2, as measured by WB in Bel-7402 cells. **d** PCBP2 boosted ubiquitination of YAP and TFCP2, as measured by co-IP in Bel-7402 cells. **e** CCT3 regulated ubiquitination of YAP and TFCP2 in a PCBP2-dependent manner, as measured by co-IP in Bel-7402 cells with or without CCT3 overexpression. **f**, **g** CCT3 regulated the expression and activity of YAP and TFCP2 via PCBP2, as measured by WB (**f**) and a pUAS-LUC/TEAD-Gal4 reporter system (**g**), respectively, in Bel-7402 cells. Images of RIP and WB are representative of threee independent experiments. **p < 0.01 indicate statistical significance. The data were analysed by a one-way ANOVA test from three independent experiments

The ubiquitination of proteins is usually associated with ubiquitin-associated proteins. To identify ubiquitin-associated proteins that simultaneously regulate YAP and TFCP2, we performed MS to screen such proteins in the immune precipitates that were immunoprecipitated by anti-YAP and anti-TFCP2 antibodies, and 288 proteins met this requirement. Subsequently, ubiquitin-associated proteins were predicted from 288 proteins using UniProt software, and five proteins, including HSPA8, p53, PCBP2, PSMA4 and VCP, were identified (Fig. 4a). However, only PCBP2 acts as an adaptor protein to be directly associated with ubiquitination^30,31^, while the remaining four proteins are either target proteins or have no effect on ubiquitination. Therefore, we chose PCBP2 in the following study.

In addition to regulating ubiquitination, PCBP2 acts as an RNA-binding protein to regulate RNA. Here, we tried to rule out such a function of PCBP2 to regulate TFCP2 and YAP. PCBP2 has been reported to bind with α-globin mRNA^32,33^. Via RNA-IP for immunoprecipitation of PCBP2, the binding of α-globin mRNA was observed; however, YAP and TFCP2 mRNA were not detected, even when CCT3 was overexpressed (Fig. 4b), suggesting that PCBP2 might not act as an RNA-binding protein to regulate YAP and TFCP2 under the control of CCT3.

Next, we tested the effects of PCBP2 on YAP and TFCP2. In both Bel-7402 and SMMC-7721 cells, PCBP2 negatively regulated YAP and TFCP2 (Fig. 4c and Supplementary Fig. S5A). In contrast to CCT3, PCBP2 reduced half-life (Supplementary Fig. S5B), and boosted ubiquitination of YAP and TFCP2 (Fig. 4d).

Then, we investigated the roles of CCT3 and PCBP2 on YAP and TFCP2. Compared to the control, CCT3-reduced ubiquitination of YAP and TFCP2 was blocked in Bel-7402 and SMMC-7721 cells with PCBP2 knocked down, whereas it was reversed when PCBP2 was simultaneously overexpressed (Fig. 4e and Supplementary Fig. S5C). Expectedly, knocking down PCBP2 prevented CCT3 to further increase YAP, TFCP2 and their targets (FYB, BICC1 and PDE3A) expression, whereas restoration of PCBP2 rescued such effects in both Bel-7402 and SMMC-7721 (Fig. 4f and Supplementary Fig. S5D). The data from the TEAD reporter system also supported that CCT3-induced YAP activity is PCBP2-dependent (Fig. 4g). Interestingly, CCT3 decreased PCBP2 expression (Fig. 4f and Supplementary Fig. S5D).

We hypothesised that PCBP2 is an E3 ligase to YAP and TFCP2 and employed a ubiquitin conjugating kit to screen possible E2 conjugating enzymes (E2) to couple with PCBP2. If PCBP2 is a genuine E3 ligase, the in vitro ubiquitination system provided by the kit will work with the aid of the appropriate E2, purified PCBP2, YAP and TFCP2. Twenty-six major human E2 proteins were screened; however, no E2 cooperated with PCBP2 and successfully ubiquitinated YAP and TFCP2 in vitro (Supplementary Fig. S5E), suggesting that PCBP2 might not be an E3 ligase.

### The PCBP2-YAP interaction was critical for CCT3 to regulate YAP and TFCP2

**Fig. 5:**
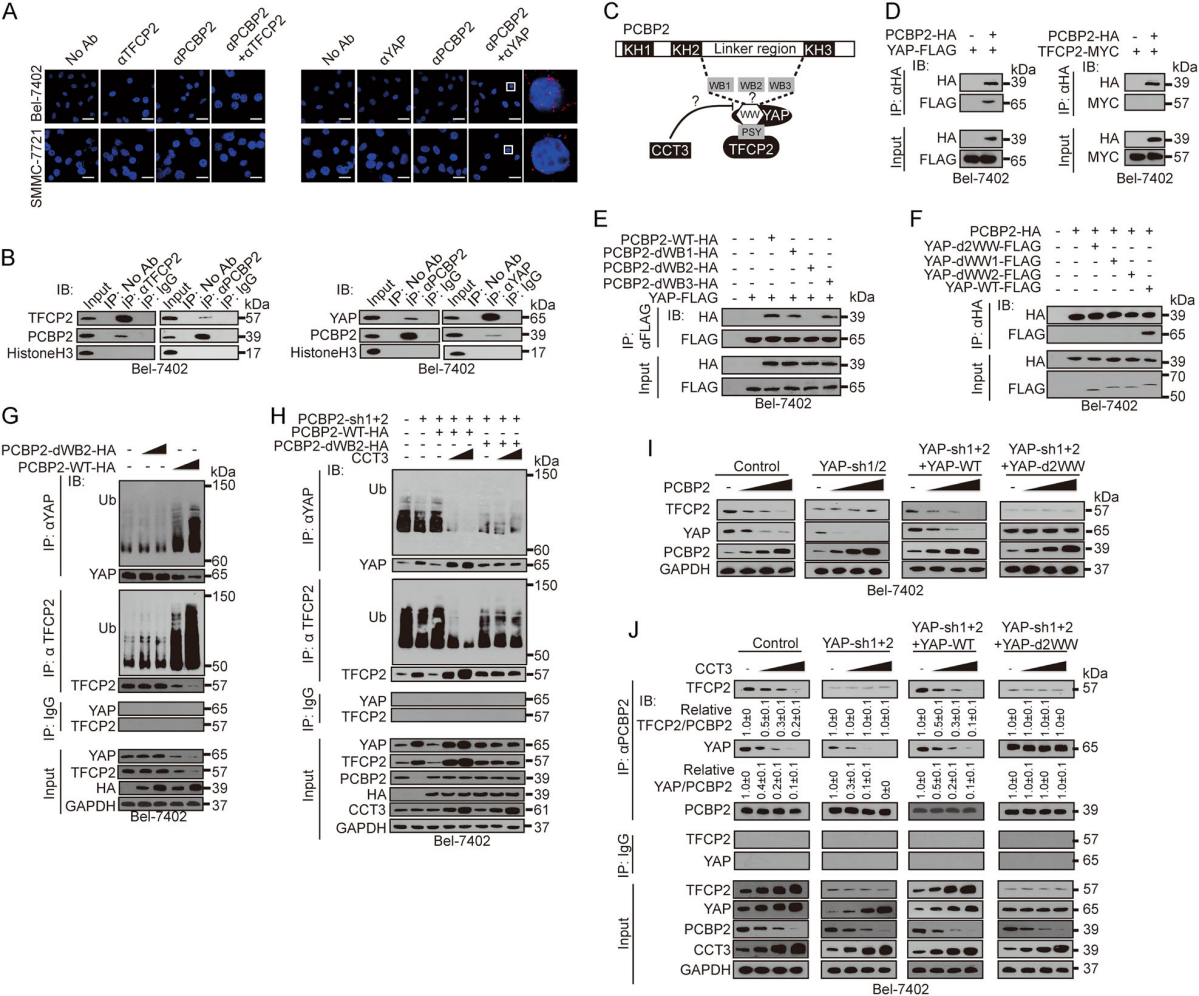
CCT3 blocked the interaction between PCBP2 and YAP/TFCP2. **a** PCBP2 directly interacted with YAP but not with TFCP2, as evaluated by PLA in Bel-7402 and SMMC-7721 cells. Scale bar, 50 μm. **b** YAP, TFCP2 bond with PCBP2, as measured by co-IP in Bel-7402 cells. **c** Schematic presentation of the hypothesis that CCT3 might prevent YAP/TFCP2 from interaction with PCBP2. **d** Interaction between exogenous PCBP2 and YAP, but not between PCBP2 and TFCP2, was identified by co-IP in Bel-7402 cells. **e** The WB2 motif was essential for the interaction between PCBP2 and YAP, as measured by co-IP in Bel-7402 cells. d, deletion. **f** The PCBP2-YAP interaction replied on the two WW domains in YAP, as measured by co-IP in Bel-7402 cells. d, deletion. **g** The WB2 motif was critical for ubiquitination of YAP and TFCP2. **h** The WB2 motif of PCBP2 was essential for CCT3-mediated suppression of ubiquitination of YAP and TFCP2, as measured in Bel-7402 cells with or without expressing increasing concentrations of CCT3. **i** The two WW domains of YAP were prerequisite for suppression of TFCP2 by PCBP2, as measured in control cells, Bel-7402 cells with YAP knocked down with or without simultaneous expressing exogenous YAP-WT/YAP-d2WW. **j** The two WW domains of YAP were essential for CCT3-induced disassociation of PCBP2 from YAP/TFCP2, as measured by co-IP in Bel-7402 cells. To clearly identify the interaction between PCBP2, TFCP2 and YAP, we artificially adjusted the concentrations of PCBP2 to the same level in the immune precipitates that were immunoprecipitated by the anti-PCBP2 antibodies. Images of WB are representative of three independent experiments

Next, we investigated the molecular mechanism underlying how CCT3 regulates YAP and TFCP2 via PCBP2. Using a protein ligation assay (PLA), which was used to find direct interactions between proteins, we found that PCBP2 directly interacted with YAP but not with TFCP2 (Fig. 5a). PCBP2 has three KH domains. Between KH2 and KH3, a linker region with three WW domain-binding (WB) motifs plays critical roles in controlling ubiquitination of targets containing the WW domain^30^. YAP has WW domains, while TFCP2 contains only one PSY motif^20^ (Fig. 5c). Does YAP directly interact with PCBP2 via the WB-WW interaction (Fig. 5c)? To answer this question, we co-expressed exogenous PCBP2-HA and YAP-FLAG or PBCP2-HA and TFCP2-MYC to avoid potential interference by endogenous proteins. Expectedly, an interaction between PCBP2 and YAP, but not between PCBP2 and TFCP2, was observed (Fig. 5d). Furthermore, the WB2, but not the WB1 and WB3 motifs, was essential for PCBP2-YAP binding (Fig. 5e). To verify whether the two WW domains in YAP are also critical for the PCBP2-YAP interaction, we separately or simultaneously deleted the WW1 and WW2 and yielded the YAP-dWW1, YAP-dWW2 and YAP-d2WW mutants. All the mutants were unable to interact with PCBP2 (Fig. 5f), suggesting that the two WW domains are equally important for the interaction with PCBP2. Collectively, the 2WW-WB2 interactions are essential for the PCBP2-YAP interaction.

Is WB2 motif prerequisite for PCBP2-mediated ubiquitination? Compared to the dose-dependent increase in the ubiquitination of YAP and TFCP2 by WT, PCBP2-dWB2 was unable to do so (Fig. 5g). To test whether the WB2 motif is involved in the regulation of YAP and TFCP2 by CCT3, we knocked down endogenous PCBP2, followed by expression of either exogenous WT- or PCBP2-dWB2. CCT3 dose-dependently reduced the ubiquitination of YAP and TFCP2 when WT was expressed; however, these effects were diminished when PCBP2-dWB2 was expressed (Fig. 5h).

Although TFCP2 was unable to directly interact with PCBP2 (Fig. 5a), TFCP2 indeed bond with PCBP2 (Fig. 5b). YAP interacts with TFCP2 via WW-PSY interactions^20^. Does PCBP2 regulate TFCP2 via YAP (Fig. 5c)? In control cells, overexpressing PCBP2 parallel decreased YAP and TFCP2 dose-dependently. However, the regulation of TFCP2 was blocked when YAP was depleted (Fig. 5i). Furthermore, such effects could only be rescued by overexpressing WT, but not YAP-d2WW (Fig. 5i), further supporting that YAP, especially its two WW domains, is required for PCBP2 to regulate TFCP2.

Next, we explored the roles of CCT3 in controlling TFCP2-PCBP2 and YAP-PCBP2 interactions. In control cells, similar to the YAP-PCBP2 interaction, the TFCP2-PCBP2 interaction was parallel and negatively controlled by CCT3 dose-dependently (Fig. 5j). However, in cells with YAP knockdown, such effects were blocked. Expectedly, the effects were restored when YAP-WT was expressed, and YAP-d2WW failed to do so (Fig. 5j). These results suggest that CCT3 also regulates the TFCP2-PCBP2 interaction via YAP.

Collectively, PCBP2 exerts its negative roles on YAP via direct WB-WW interactions and on TFCP2 indirectly via the aid of YAP. CCT3 abolishes PCBP2 to interact with YAP and TFCP2, thus preventing YAP and TFCP2 from being ubiquitinated.

### CCT3, YAP, TFCP2 and PCBP2 in transformative phenotypes

**Fig. 6:**
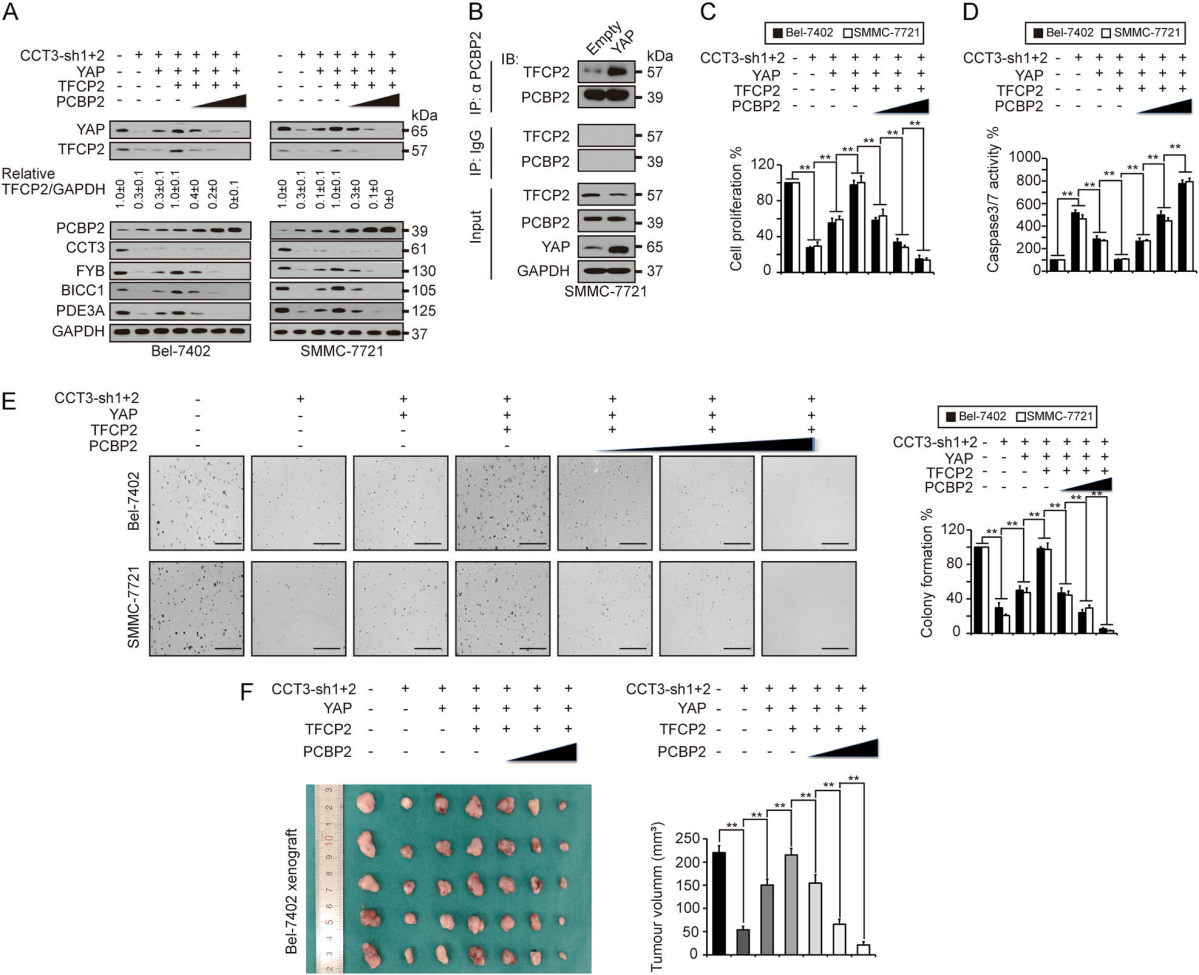
The functions of YAP, TFCP2 and PCBP2 in CCT3 knockdown-suppressed transformative phenotypes. **a** Representative WB images of YAP/TFCP2 and their targets in control cells, Bel-7402 and SMMC-7721 cells with CCT3 knocked down in the presence or absence of YAP, TFCP2 or PCBP2 overexpression. **b** YAP boosted TFCP2 binding to PCBP2, as measured by co-IP in SMMC-7721 cells. **c**–**e** The cell proliferation capacity (**c**), caspase 3/7 activity (**d**) and colony formation capacity (**e**) were evaluated in Bel-7402 and SMMC-7721 cells with CCT3 knockdown in the presence or absence of YAP, TFCP2 or PCBP2 overexpression. **f** CCT3 knockdown-reduced in vivo tumour growth could be reversed by YAP and TFCP2, which were blocked by increasing PCBP2. *n* = 5/group. The data were analysed from three independent experiments (except Fig. 6f) using a one-way ANOVA test. **p < 0.01 indicates statistical significance

Here, we tested whether the relationship among CCT3, YAP, TFCP2 and PCBP2 is functional in maintaining transformative phenotypes in liver cancer cells. To address this, we prepared cells under different conditions (Fig. 6a). In cells with CCT3 knockdown, YAP and TFCP2 were downregulated, while PCBP2 was upregulated (Fig. 6a). Overexpressing YAP only reversed itself, but instead slightly reduced TFCP2 in SMMC-7721 cells (Fig. 6a). Interestingly, overexpressing YAP recruited TFCP2 to PCBP2 (Fig. 6b), this might increase the efficiency of PCBP2 to degrade TFCP2, which might explain why YAP reduces TFCP2 in SMMC-7721 cells. However, in cells overexpressing YAP, overexpressing TFCP2 rescued itself and enhanced YAP simultaneously (Fig. 6a), further supporting that TFCP2 reinforces YAP^20^. Expectedly, increasing PCBP2 reduced YAP and TFCP2 dose-dependently, even when YAP and TFCP2 were overexpressed (Fig. 6a). Furthermore, the expression of FYB, BICC1 and PDE3A was correlated with YAP and TFCP2 (Fig. 6a).

Then, we investigated whether these cells display different transformative phenotypes. Knocking down CCT3 significantly suppressed cell proliferation (Fig. 6c), colony formation in soft agar (Fig. 6e), and tumour growth in mice models (Fig. 6f), while elevated caspase 3/7 activity (Fig. 6d). However, these effects were reversed by YAP overexpression and enhanced by simultaneous TFCP2 overexpression (Fig. 6c–f), suggesting that YAP and TFCP2 might act downstream of CCT3. Similar to the regulation of YAP and TFCP2 expression (Fig. 6a), PCBP2 suppressed the enhanced transformative phenotypes resulting from co-overexpression of YAP and TFCP2 dose-dependently (Fig. 6c–f). Together, the CCT3-YAP-TFCP2-PCBP2 axis might be critical for tumourigenesis in liver cancer.

### Clinical value of CCT3

**Fig. 7:**
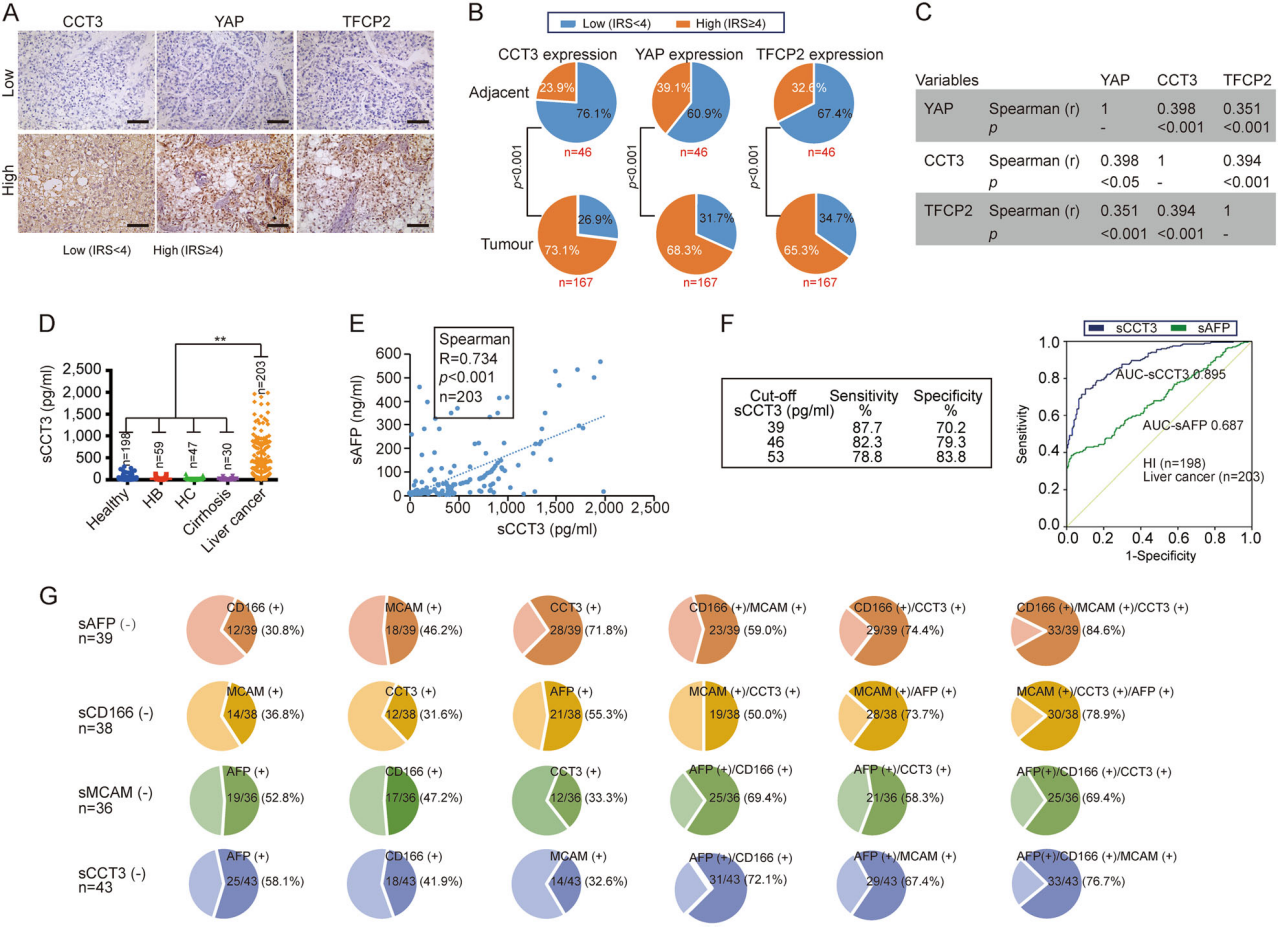
Clinical significance of CCT3 in liver cancer. **a** Representative IHC images of liver cancer tissues with low and high expression of CCT3, YAP and TFCP2. Scale bar, 500 μm. **b** Pie charts showing the percentages of cases with low and high expression of CCT3 (left), YAP (middle) and TFCP2 (right) in adjacent non-tumour and tumour specimens from liver cancer patients. The differences between adjacent non-tumour and tumour specimens were analysed by z test. **c** Table showing the correlation among CCT3, YAP and TFCP2 in 167 liver cancer specimens. The data was analysed by Spearman’s correlation test. **d** sCCT3 in serum samples from healthy individuals, patients with hepatitis B (HB), hepatitis C (HC), cirrhosis and liver cancer. The data was analysed by one-way ANOVA. **p < 0.01. **e** The correlation between sAFP and sCCT3 in serum samples from 203 liver cancer patients, as analysed by Spearman’s correlation test. **f** ROC curves for sCCT3 and sAFP for the discrimination of patients with liver cancer (*n* = 203) from healthy individuals (HI, *n* = 198). The cutoff value, sensitivity and specificity for sCCT3 are also listed. **g** Combination usage of sCD166, sMCAM, sCCT3 and sAFP increased sensitivity for the diagnosis of liver cancer. Pie chart showing the positive percentage of single or combination of sCD166, sMCAM, sCCT3 and sAFP in sAFP, sCD166, sMCAM and sCCT3 false negative liver cancer serum samples

To assess the clinical relevance of our findings, a tissue microarray assay (TMA) containing 213 cases (46 non-tumour adjacent and 167 tumour from liver cancer patients) was performed to evaluate the expression of YAP, TFCP2 and CCT3. By calculating the immunoreactivity score (IRS) for YAP, TFCP2 and CCT3 in each specimen, we classified the specimens into two groups [i.e., low (with IRS < 4) and high (with IRS ≥ 4)] for each protein (Fig. 7a, b). In tumour specimens, more cases were classified into the high group compared to the low group for all three proteins (*p* < 0.001). In contrast, in adjacent non-tumour specimens, more cases were classified into the low group compared to the high group (*p* < 0.001) (Fig. 7b). In addition, unlike adjacent non-tumour specimens, tumour specimens with higher CCT3 often had higher YAP/TFCP2, and vice versa (Fig. 7a, b). Spearman’s rank correlation coefficient analysis was then performed according to the IRS to analyse the data from 167 tumour specimens, and significant correlations among YAP, CCT3 and TFCP2 were revealed (Fig. 7c), suggesting that the CCT3-YAP-TFCP2 loop might be important in liver cancer.

Is CCT3 a serum liver cancer biomarker? Seral-CCT3 (sCCT3) was specifically elevated in liver cancer compared to that in healthy individuals, hepatitis B (HB), HC, and cirrhosis (Fig. 7d). Additionally, a significant correlation between sAFP and sCCT3 (Spearman, *R* = 0.734, *p* < 0.001) was revealed (Fig. 7e). A receiver operating characteristic (ROC) curve analysis was performed in healthy individuals (*n* = 198) and liver cancer patients (*n* = 203). The area under the ROC curve (AUC) indicated that sCCT3 might be a better screening biomarker than sAFP (Fig. 7f). The best cutoff value of sCCT3 for predicting liver cancer from healthy individuals is 53 pg/ml, with sensitivity and specificity of 78.8% and 83.8%, respectively (Fig. 7f).

Even for sAFP, its sensitivity for liver cancer is 49–71%^34^. Can sCD166, sMCAM and sCCT3 improve the sensitivity of sAFP? In sAFP false negative serum (*n* = 39), 30.8% (12/39) were sCD166 positive, 46.2% (18/39) were sMCAM positive, and 71.8% (28/39) were sCCT3 positive (Fig. 7g). Notably, the combination usage of sMCAM and sCCT3 significantly increased the true positive rate that resulted from the single usage of sCD166, with the highest when sCD166, sMCAM and sCCT3 were combined applied (Fig. 7g), suggesting combination usage of sCD166, sMCAM and sCCT3 supplements the inadequacies of sAFP. Similarly, the false negatives of sCD166, sMCAM and sCCT3, respectively, could be significantly improved by the other three biomarkers (Fig. 7g), indicating that this panel is useful to elevate sensitivity for liver cancer diagnosis.

## Discussion

**Fig. 8:**
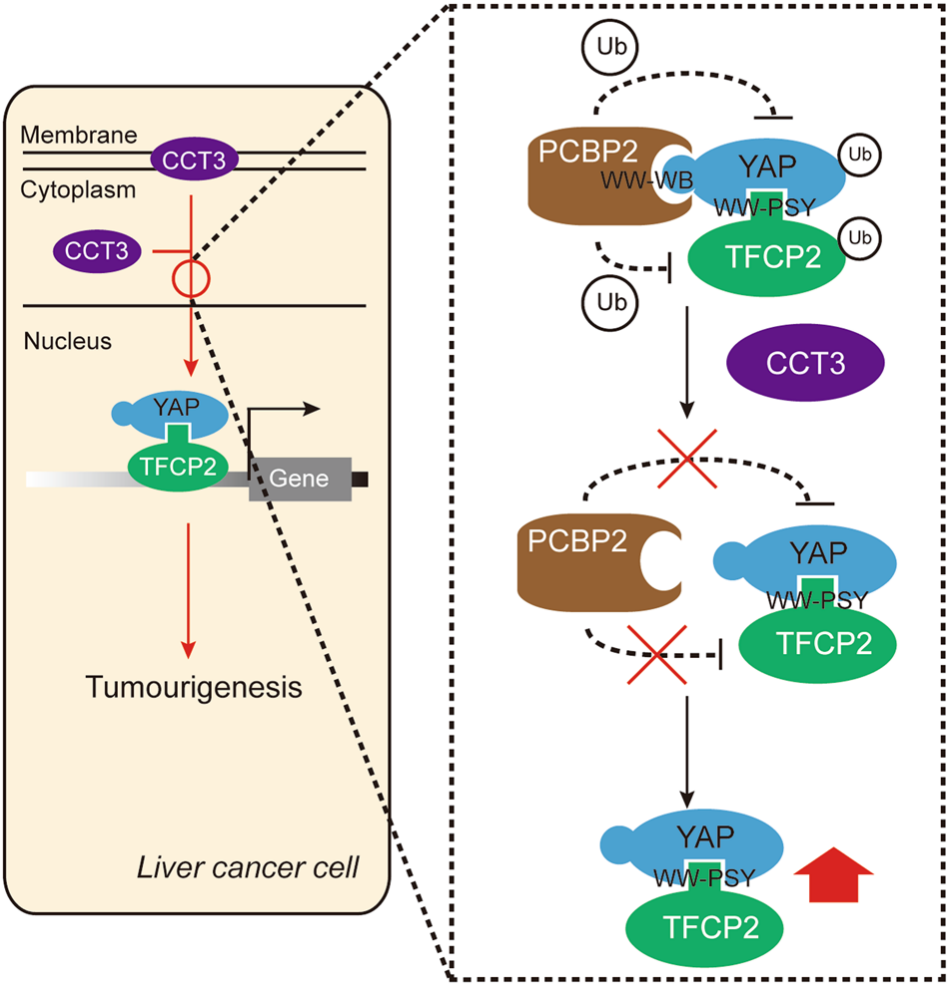
Schematic diagram of the mechanism underlying CCT3 stimulation of YAP and TFCP2 in liver cancer. Briefly, PCBP2 interacts with YAP directly via the WW-WB interaction and interacts with TFCP2 indirectly via the aid of YAP through the WW-PSY interaction. The ubiquitination of YAP and TFCP2 was mediated by their interactions with PCBP2. However, CCT3 prevents PCBP2 from binding with YAP and TFCP2, thereby reducing their ubiquitination and increasing their stability. Via such effects, the protumourigenic functions of YAP and TFCP2 in liver cancer are enhanced

There are several Hippo-independent ways of YAP regulation^28,35,36^. In liver cancer, YAP can be regulated in a LATS- and MST-independent manner^16,17,20,26^. Interestingly, genetic depletion of components belonging to Hippo is not sufficient to result in tumour formation^37^. Here, we found that CCT3 stimulates YAP expression via a Hippo-independent mechanism (Fig. 8), providing additional evidence that Hippo-independent signalling is equal important to that of canonical Hippo signalling.

PCBP2 plays important roles in a series of cancers, and its functions are based on RNA binding^38–42^. Although PCBP2 affects hepatitis C virus (HCV) RNA synthesis^43^ and HCV-induced innate immune responses^44^, the exact role of PCBP2 in liver tumourigenesis remains unclear. Here, we uncovered another tumour suppressive role of PCBP2 to reduce the protein stability of YAP and TFCP2 in liver cancer. Apart from its role in binding with nucleic acids, PCBP2 interacts and mediates the degradation of target proteins via recruiting other ubiquitin E3 ligases^30,31,45^. To date, PCBP2 has not yet been proven to be a genuine ubiquitin E3 ligase. However, PCBP2 is a prerequisite for the function of HECT ubiquitin E3 ligases^30^. Unfortunately, our MS data were unable to define any potential E3 ligase to co-interact with YAP and TFCP2, which might be due to the low abundance of the protein or the fact that the E3-ligase-YAP/TFCP2 interaction is not a direct interaction. βTrCP is not involved in the PCBP2-mediated regulation of YAP/TFCP2. This might be because βTrCP belongs to the F-box protein family but not belongs to the HECT family. In fact, the human HECT E3 family consists of 28 members^46^, which all have the possibility to interact with PCBP2. The link among YAP/TFCP2, PCBP2 and E3 ligase should be thoroughly investigated.

Here, inhibiting CCT3 has been proven to impair transformative phenotypes in liver cancer cells. Furthermore, CT20p, a therapeutic peptide, displays selective cytotoxicity to cancer progression through inhibiting CCT proteins^47^, suggesting that CCT-related cancers are treatable. Importantly, we have linked CCT3 to YAP; therefore, targeting CCT3 might be effective in treating YAP-associated liver cancer.

AFP is commonly used to assist in the diagnosis of liver cancer. However, AFP does not yield satisfactory outcomes, particularly in AFP-negative HCC^48^. We found that the true positive rate of liver cancer can be increased by the combined usage of previously discovered CD166 and MCAM, current identified CCT3 and traditional AFP. This panel of biomarkers exhibits potential value for liver cancer screening. However, the conclusion was limited because it is not a multicentre study. It is also worth noting that 15.4–30.6% of patients with liver cancer cannot be well diagnosed even when CD166, MCAM, CCT3 and AFP were combined used (Fig. 7g), this led us to discover more useful biomarkers to overcome this problem.

Taken together, in this paper, CCT3 has been identified as an upstream trigger to increase the protein stability of YAP and TFCP2 in liver cancer cells. Importantly, CCT3 can also be regarded as a potential therapeutic target and serum biomarker, thus providing new ways to treat and diagnose liver cancer.

## Materials and methods

### Cell culture and vectors

The liver cancer cell lines Bel-7402 and SMMC-7721 were cultured in DMEM. Cells were treated with CHX [Solarbio (Shanghai, China), final concentration 50 mM] for the indicated time before harvest for further analysis. TEAD-Gal4/pUAS-LUC, plasmids expressing YAP, TFCP2 and βTrCP, and lentiviral-based shRNA targeting YAP (sh1&2) and TFCP2 (sh1&2) were obtained from our previous studies^16,20,26^. The plasmids expressing CCT3 and PCBP2 and lentiviral-based shRNA against CCT3 (sh1&2) and PCBP2 (sh1&2) were purchased from Biolink LTD (Shanghai, China) and GE Healthcare Pte. LTD (Singapore), respectively. The siRNAs targeting LATS1, LATS2, MST1 and MST2 were designed as follows: siLATS1: GAGCUGGAAAGGUUCUAAAUU; siLATS2: GUUCGGACCUUAUCAGAAAUU; siMST1: GACCAAAGGUACGGGUAAUUU; siMST2: GCCCAUAUGUUGUAAAGUAUU. The plasmids expressing YAP-dWW1-FLAG, YAP-dWW2-FLAG, YAP-d2WW-FLAG and YAP-WT-FLAG were constructed as we previously reported with the HA-tag replaced by the FLAG-tag^20^. The PCBP2-WT-HA expressing plasmid was constructed using pcDNA3.1 as the backbone. The PCBP2-dWB1-HA, PCBP2-dWB2-HA, and PCBP2-dWB3-HA plasmids were constructed using the overlapping PCR with the primers listed below: WT-F: 5′ GCGCGGATCCATGGACACCGGTGTGATTGAAGG 3′, WT-R: 5′ GCGCCTCGAGCTAAGCGTAGTCTGGGACGTCGTATGGGTAGCTGCTCCCCATGCCACCCG 3′;

dWB1-F: 5′ AAGGGCGTGACCATCCCGTACCGGC 3′

dWB1-R: 5′ GATGGTCACGCCCTTAGTCTCCAACATGACCACGCAGATC 3′;

dWB2-F: 5′ ATCTTTGCAGGTGGTCAGGACAGGT 3′

dWB2-R: 5′ ACCACCTGCAAAGATCTTGGGCCGGTACGGGATGGTCACG 3′;

dWB3-F: 5′ GTGAAAGGCTATTGGGCAGGTTTGG 3′

dWB3-R: 5′ CCAATAGCCTTTCACAATGCCACTGAATCCGGTGTTGCCA 3′.

### Immunofluorescence, Western blotting and immunohistochemistry

The protocols of Immunofluorescence (IF), Western blotting (WB) and immunohistochemistry (IHC) are conventional ones, which are available elsewhere. The primary antibodies used for IF were anti-YAP [Cell signalling technology (CST), Boston, MA, USA, #12395], anti-TFCP2 (CST, #80784), anti-CCT3 (Abcam, Hong Kong, China, #ab174255, or Santa Cruz biotechnology, Santa Cruz, CA, USA, #sc-271336). The slides were incubated with Alexa Fluor®‑488/555 fluorescent conjugated secondary antibodies (CST, #4408 or CST, #4413) before being mounted with ProLong® Gold antifade reagent with DAPI (Molecular Probes, Eugene, OR, USA). The potential protein-protein interactions were observed using an LSM 800 Confocal Microscope (Carl Zeiss AG, Oberkochen, Germany). For WB, cellular nuclear and cytosol extracts were prepared using a kit from Active Motif (Carlsbad, CA, USA). The primary antibodies used for WB include: anti-CCT3 (Abcam, #ab174255, or Santa Cruz, #sc-271336), anti-CCT2 (Abcam, #ab92746, or Santa Cruz, #sc-373769), anti-FLAG (CST, #8146 or #2368), anti-HA (CST, #2367 or #3724), anti-MYC (CST, #2276 or #2278), anti-YAP (Abcam, #ab52771, or CST, #12395), anti-TFCP2 (Abcam, #ab180033, or CST, #80784), anti-FYB (Abcam, #ab201667), anti-PDE3A (Abcam, #ab169534), anti-BICC1 (Abcam, #ab175955), anti-Ub (CST, #3933, or CST, #3936), anti-PCBP2 (Abcam, #ab184962, or Santa Cruz, #sc-101136), anti-p-YAP^S127^ (Abcam, #ab4911), anti-p-LATS1^T1079^/ LATS2^T1041^ (Abcam, #ab4911), anti-LATS1 (CST, #3477), anti-LATS2 (CST, #5888), anti-p-MST1^T183^/MST2^T180^ (Abcam, #ab79199), anti-MST1 (CST, #3682), anti-MST2 (CST, ##3952), anti-Histone H3 (CST, #4499), anti-βtubulin (Abcam, #ab179513), anti-βTrCP (CST, #4394, or Abcam, # ab118006), anti-AGPAT1 (Abcam, #ab67018), anti-LAMTOR1 (CST, #8975), anti-CCT6A (Abcam, #ab110905, or Santa Cruz, #sc-271734), anti-GAPDH (CST, #5174) or anti-5Sa (Abcam, #ab137109). The membranes were incubated with secondary antibodies conjugated with horseradish peroxidase (CST, #7074 or #7076) and visualised using Pierce ECL Western Blotting Substrate (Thermo Fisher Scientific, Inc. Waltham, MA, USA). The antibodies used for IHC are listed below: anti-CCT3 (Abcam, #ab174255), anti-YAP (CST, #12395), and anti-TFCP2 (CST, #80784). The specimens were scored semiquantitatively on the basis of the well-established immunoreactivity score system (IRS). The IRS is calculated by multiplying the score for the percentage of positive cells (4, > 80%; 3, 51–80%; 2, 10–50%; 1, < 10%; 0, 0%) and the staining intensity (3, strong; 2, moderate; 1, mild; and 0, no staining), which results in IRS scores between 0 and 12. Specimen with IRS ≥ 4 were considered high expression, while IRS < 4 was considered low expression.

### Cell proliferation, Caspase 3/7 activity, soft agar colony formation assay, and quantitative RT-PCR

Cell proliferation was measured by an MTT-based assay, Caspase 3/7 activity was measured by a Caspase 3/7 Glo luciferase reagent (Promega, Madison, WI, USA) and colony formation capacity was evaluated by an anchorage-independent soft-agar colony-formation assay. cDNA synthesis was conducted using the PrimeScript™ RT Master Mix (Takara, Dalian, China). GAPDH was used as an internal control, and quantitative RT-PCR (qPCR) was performed using ABI7900 with qPCR reagent (Kapa, Wilmington, MA, USA). The primers used for qPCR are listed below: FYB-F: 5′ CTCCACCAAAACCCAACAGACC’, FYB-R: 5′ GTTGTGATGGGTGAGATGCTGG’; BICC1-F: 5′ TCCCGAATGTATGGTGCTACTG 3′, BICC1-R: 5′ TGATGTTGCTCCCATTTCGACC 3′; PDE3A-F: 5′ AAGCCCAGAGTGAATCCCGTC 3′, PDE3A-R: 5′ ACTCGTCTCAACAAGCCAGGAGG’; YAP-F: 5′ CCTCGTTTTGCCATGAACCAG3’, YAP-R: 5′ GTTCTTGCTGTTTCAGCCGCAG3’; TFCP2-F: 5′ ATGGCCCGAGATCACGTATG 3′, TFCP2-R: 5′ TCCTGAGGTGTGGTTGTTGG 3′; GAPDH-F: 5′ ATCATCCCTGCCTCTACTGG 3′, GAPDH-R: 5′ GTCAGGTCCACCACTGACAC 3′.

### Co-immunoprecipitation

Co-immunoprecipitation (co-IP) was performed as described previously^20,26,49^. The reagents used included protein A/G-Sepharose (Life sciences, Oslo, Norway) and Western/IP lysis buffer (Beyotime, Haimen, China). The antibodies used for detecting the interactions for endogenous and exogenous proteins were anti-YAP (CST, #12395), anti-TFCP2 (Abcam, #ab180033), anti-PCBP2 (Abcam, #ab184962), anti-CCT3 (Abcam, #ab174255), anti-βTrCP (CST, #4394), anti-IgG (CST,#5415,or CST, #3900), anti-AGPAT1(Abcam, #ab235328), anti-LAMTOR1 (CST, #8975), anti-CCT6A (Abcam, #ab110905), anti-CCT2 (Abcam,#ab92746), anti-FLAG (CST, #8146), anti-HA (CST, #2367), or anti-MYC (CST, #2276).

### Mice experiments and tissue samples

Bel-7402 cells (5 × 10^6^) under different treatments were subcutaneously injected into 8-week-old athymic nude mice (Bikai, Shanghai, China). The tumour size was measured 8 weeks after injection, and the tumour volume was calculated as 0.5 × L × W^2^, where L is length and W is width. All mouse experiments were performed according to the institutional guidelines of Shanghai Tenth People’s Hospital. Fresh tumourous and adjacent liver tissues were acquired from the Shanghai Tenth People’s Hospital under institutional approval. Informed written consent was obtained from all patients. The tissue microarray used in Fig. 7 was purchased from U.S. Biomax through the agent Alenabio (Xi’an, China).

### Protein ligation assay

Protein ligation assay (PLA) was performed using the Duolink In Situ Red Starter Kit (mouse/rabbit) (Sigma-Aldrich, St. Louis, MO, USA). Cells were seeded on glass cover slips in 24-well plates. On the second day, the cells were fixed with 4% PFA for 15 min and blocked with blocking buffer supplied by the manufacturer for 1 h. After blocking, the cells were incubated overnight at 4 °C in suitable primary antibodies. The primary antibodies used were anti-CCT3 (Abcam, #ab174255), anti-YAP (CST, #12395), anti-TFCP2 (Abcam, #ab180033) or anti-PCBP2 (Abcam, #ab184962).

### RNA immunoprecipitation

RNA immunoprecipitation (RIP) was performed according to previously reported procedures^50^. The antibodies used for RIP were anti-PCBP2 (CST, #14074). cDNA was synthesised from the co-immunoprecipitated RNA, and the results were visualised by PCR using the following primer sets: α-globin-F: 5′ CAACTTCAAGCTCCTAAGCCACT 3′, α-globin-R: 5′ CACAGAAGCCAGGAACTTGTCCA 3′; YAP-F: 5′ CAACACTGGAGCAGGATGGT 3′, YAP-R: 5′ GGTTCGAGGGACACTGTAGC 3′; TFCP2-F: 5′ ATGGCCCGAGATCACGTATG 3′, TFCP2-R: 5′ TCCTGAGGTGTGGTTGTTGG 3′.

### In vitro ubiquitination assays

Due to the complexity of the ubiquitination system, it can be challenging to determine which E2 conjugating enzyme(s) is utilised by a recently discovered or poorly characterised E3 ligase. PCBP2 is such a protein with no clear function as an E3 ligase. In this assay, we supposed PCBP2 is an E3 ligase to YAP and TFCP2 and used a ubiquitin conjugating kit (R&D systems, Minneapolis, MN, USA) to screen possible E2 to couple with PCBP2. If PCBP2 is a genuine E3 ligase, the in vitro ubiquitination system provided by the kit will work with the aid of the appropriate E2. Purified PCBP2, YAP and TFCP2 were purchased from Abnova (Taiwan, China). Twenty-six major human E2 enzymes were identified in this assay, and the positive result was visualised by WB as a shifted ubiquitinated protein.

### Mass spectrometry

To reveal the identities of the protein possibly interacting with YAP and TFCP2, the protein bands in the Coomassie Brilliant blue gel were excised and in-gel digested with trypsin. The tryptic peptide digests of the proteins were analysed using capillary electrophoresis/nano-liquid chromatography (Nano-LC) systems coupled with an electrospray ionisation and quadrupole-time-of-flight mass spectrometer (ESI-QTOF-MS, Bruker Daltonics, Leipzig, Germany). An internal MASCOT 2.4.1 server (Matrix Science, Boston, MA, USA; http://www.matrixscience.com/) using the Swiss-Prot database was used to identify peptides. MS data have been deposited in the ProteomeXchange under accession no. PXD013143 (Username: reviewer29576@ebi.ac.uk, password: NoEt1GFR).

### Bioinformatics

Protein subcellular localisation was acquired from the human protein atlas (THPA) database (https://www.proteinatlas.org/search) and the UniProt database (http://www.uniprot.org/). Proteins related to ubiquitination were screened from the UniProt database. The mRNA expression elevated in liver cancer was obtained from GEO: GSE62232, GSE14520, GSE64041 and GSE45267. The results of CCT3 mRNA expression and survival analysis were based upon data generated by the TCGA database (https://portal.gdc.cancer.gov/). Patient cohort and mRNA data were obtained from the TCGA level 3 data. The downloaded clinical data were matched to the mRNA expression profile. Therefore, some patients were excluded, such as those missing mRNAs expression level, without follow-up data, or without most clinical information. The mRNA expression processing method is log2 (*x* + 1). Differentially expressed mRNAs between cancer and normal tissues were screened by *t*-test in the R software (*p* < 0.05). The data were dichotomised into high-level and low-level groups, followed by the Kaplan–Meier survival analysis to determine whether they had clinical outcomes.

### Serum samples

Serum samples were obtained from patients who were diagnosed with primary liver cancer (mean age ± SD, 64.43 ± 8.62 years; male: female ratio, 1.65:1), hepatitis B (mean age ± SD, 38.86 ± 6.45 years; male: female ratio, 1.27:1), hepatitis C (mean age ± SD, 56.42 ± 8.13 years; male: female ratio, 1.14:1) or cirrhosis (mean age ± SD, 58.04 ± 6.93 years; male: female ratio, 1.73:1) at Shanghai Ruijin Hospital (Shanghai, China) between May 2015 and May 2017 for this study. Serum from 198 healthy individuals (mean age ± SD, 54.64 ± 10.74 years; male: female ratio, 1.02:1) were simultaneously collected at Shanghai Tenth People’s Hospital as control samples. Informed written consent was obtained from all patients. Serum samples were collected under institutional approval. The serum was centrifuged, aliquoted and stored at −80 °C. Liver cancer patients were diagnosed using histopathological analysis. The concentrations of sAFP, sCD166, sMCAM and sCCT3 in sAFP, sCD166, sMCAM and sCCT3-negative liver cancer patients were less than 20, 311, 2400 and 53 pg/ml, respectively. Hepatitis B or C patients were confirmed through detection of more than 1 × 10^3^ copies of HBV-DNA or HCV-RNA in the serum using the kit from Kehua (Shanghai, China).

### Enzyme linked immunosorbent assay

The concentrations of sAFP, sCD166, sMCAM and sCCT3 were detected using ELISA. ELISA kits were purchased from Lichen Biotech Ltd. (Shanghai, China). Enzyme linked immunosorbent assay (ELISA) experiments were performed in strict accordance with the manufacturers’ guidelines.

### Statistical analysis

Tests to examine the differences between groups included Student’s *t* test and one-way ANOVA. The levels of mRNA expression between cancer tissues and normal tissues were analysed by *t*-test. All analyses related to patient survival were tested by Kaplan–Meier survival analysis (log-rank method). A *p* < 0.05 was regarded as statistically significant.

## Supplementary information

Supplementary Figures.S1-S5

Supplementary table. S1
