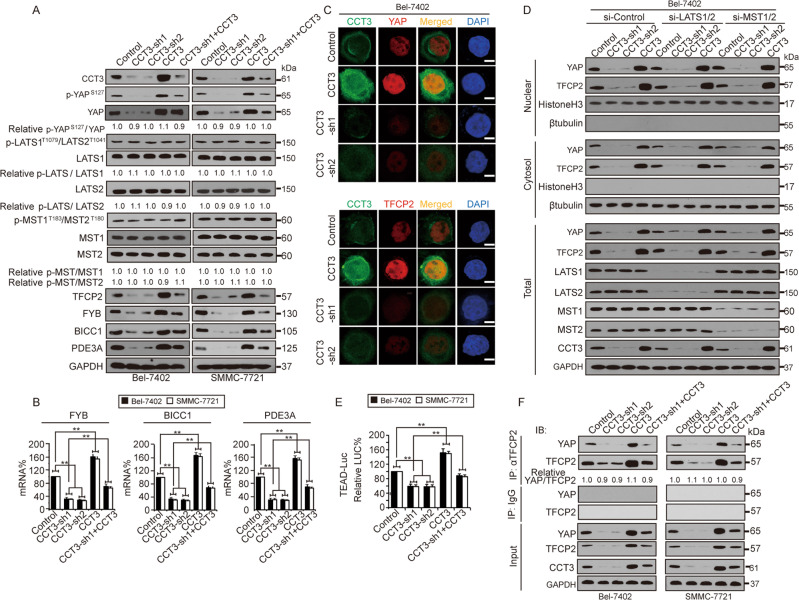# Correction: CCT3 acts upstream of YAP and TFCP2 as a potential target and tumour biomarker in liver cancer

**DOI:** 10.1038/s41419-022-05012-3

**Published:** 2022-07-12

**Authors:** Ya Liu, Xiao Zhang, Jiafei Lin, Yuxin Chen, Yongxia Qiao, Susu Guo, Yueyue Yang, Guoqing Zhu, Qiuhui Pan, Jiayi Wang, Fenyong Sun

**Affiliations:** 1 Department of Clinical Laboratory, Shanghai Tenth People’s Hospital of Tongji University, 200072 Shanghai, China; 2 Shanghai Institute of Thoracic Tumours, Shanghai Chest Hospital, Shanghai Jiaotong University, 200030 Shanghai, China; 3 Department of Clinical Laboratory, Shanghai Ruijin Hospital, Shanghai Jiaotong University, 200025 Shanghai, China; 4 School of Public Health, Shanghai Jiaotong University School of Medicine, 200025 Shanghai, China; 5 Department of Laboratory Medicine, Shanghai Children’s Medical Center, Shanghai Jiaotong University School of Medicine, 200127 Shanghai, China

**Keywords:** Tumour biomarkers, Transcriptional regulatory elements

Correction to: *Cell Death and Disease* 10.1038/s41419-019-1894-5, published online 09 September 2019

The original version of this article unfortunately contained a mistake. After the publication of this paper in Cell Death and Disease in 2019, the authors noted an error in Figure 2a, in that, the YAP blot for the SMMC-7721 cells was by mistake duplicated for the Bel-7402 cells. The correct YAP blot for Bel-7402 is now included in the figure given here. The authors would like to apologize for any inconvenience caused.