# Supplementary material for: CCT3 acts upstream of YAP and TFCP2 as a potential target and tumour biomarker in liver cancer
Source: Cell Death Dis. 2019 Sep 9;10(9):644. doi: 10.1038/s41419-019-1894-5 (PMC6733791; doi:10.1038/s41419-019-1894-5)
Supplement: Supplementary file 1 — Supplementary Figures.S1-S5 [file 41419_2019_1894_MOESM1_ESM.doc]

**Supplementary Figures. S1-5**

**Supplementary Figure S1.**

**
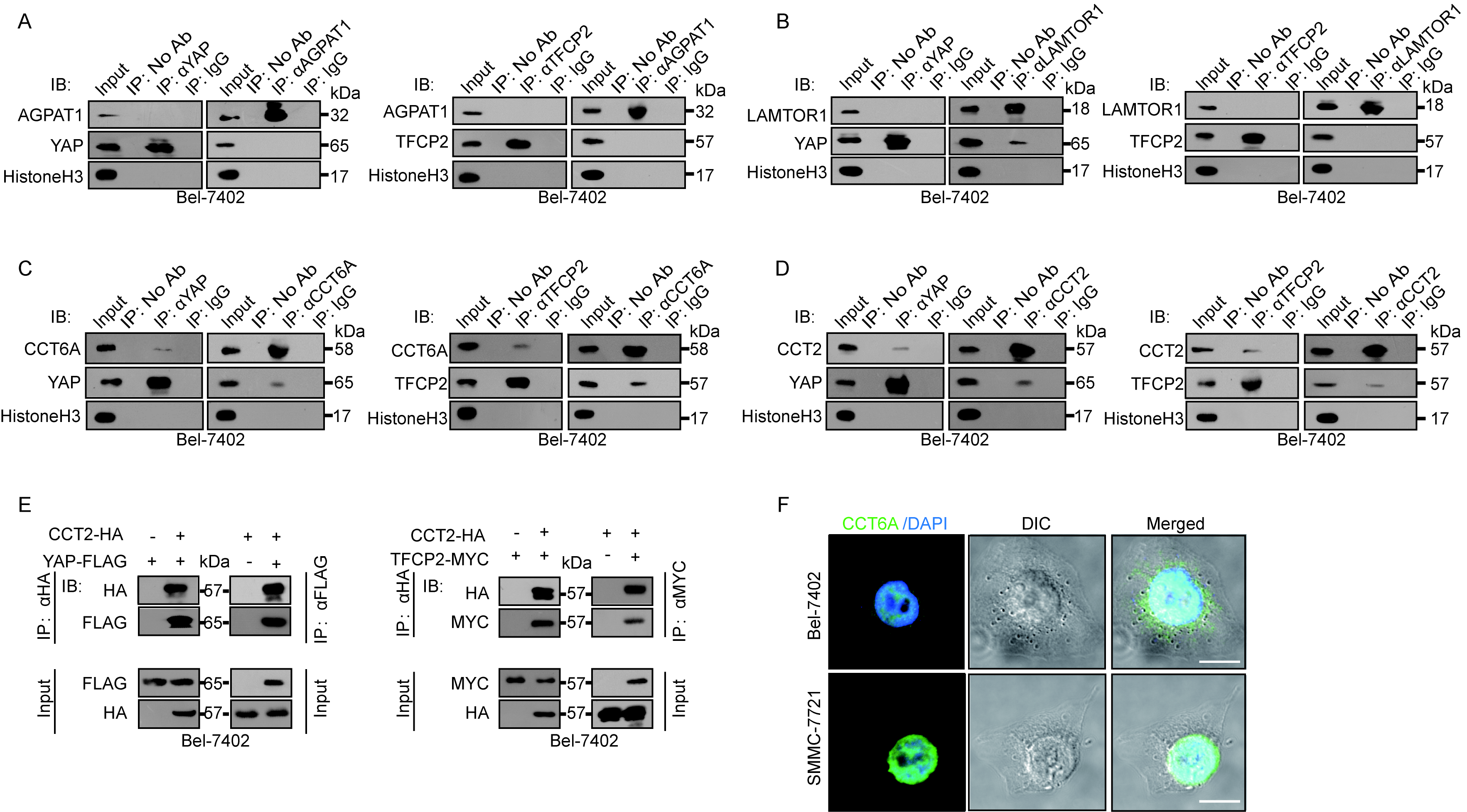
**

**Supplementary Figure S1. Verification of YAP/TFCP2-associated proteins**

(A) No obvious interaction between endogenous AGPAT1 and YAP/TFCP2 was observed in Bel-7402 cells, as measured by co-IP.

(B) No obvious interaction between endogenous LAMTOR1 and YAP/TFCP2 was observed in Bel-7402 cells, as measured by co-IP.

(C) Interaction between endogenous CCT6A and YAP/TFCP2 was observed in Bel-7402 cells, as measured by co-IP.

(D) Interaction between endogenous CCT2 and YAP/TFCP2 was observed in Bel-7402 cells, as measured by co-IP.

(E) Interaction between exogenous CCT2 and YAP/TFCP2 was observed in Bel-7402 cells, as measured by reciprocal co-IP.

(F) Subcellular localization of CCT6A in Bel-7402 and SMMC-7721 cells, as measured by IF.

Images of WB are representative ones from 3 independent experiments.

**Supplementary Figure S2.**

**
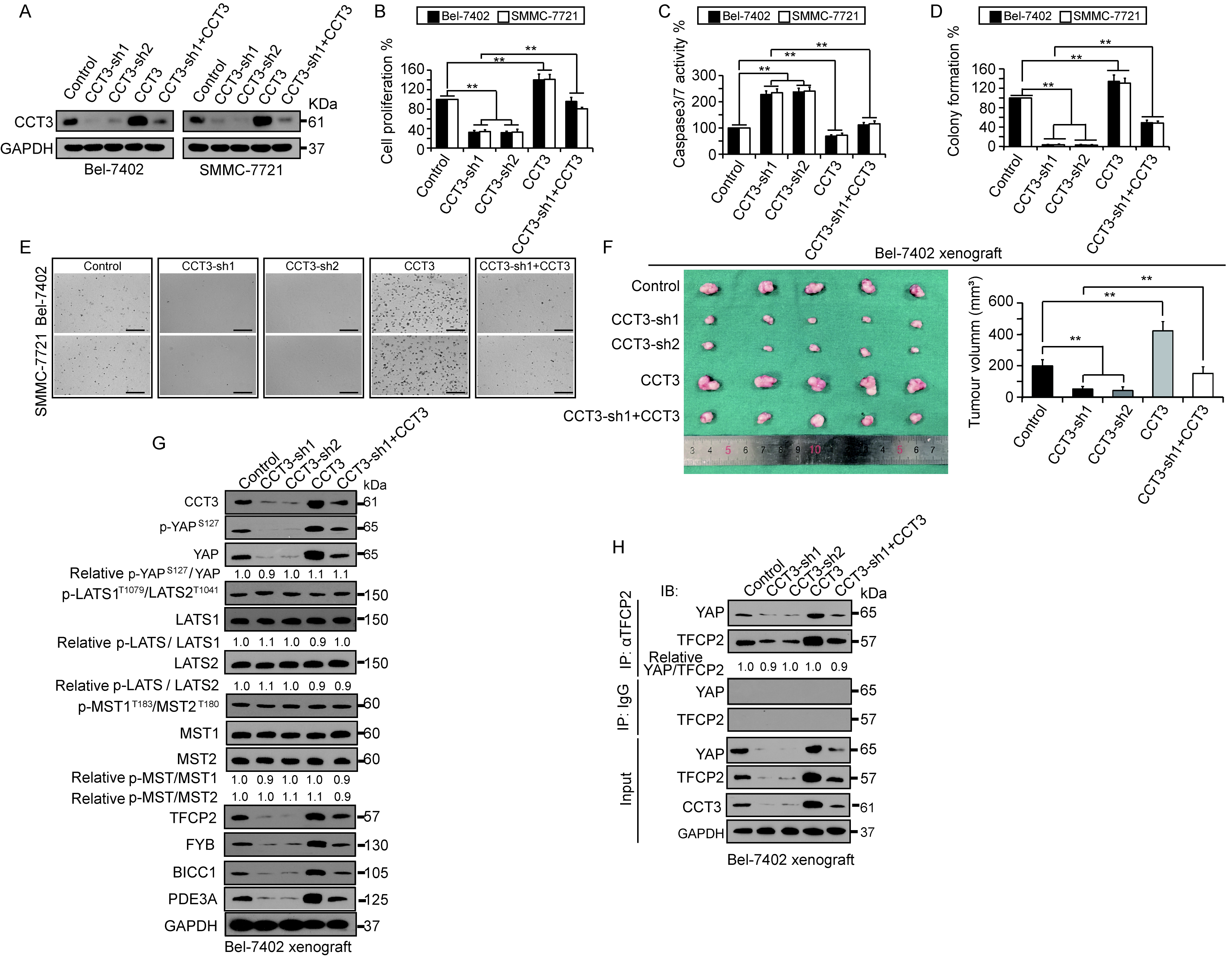
**

**Supplementary Figure S2.** **CCT3 was critical for transformative phenotypes**

(A) The representative WB images of CCT3 in control cells, Bel-7402 and SMMC-7721 cells with or without CCT3 knocked down or overexpressed.

(B-E) The cell proliferation, caspase 3/7 activity and colony formation capacity in control cells, Bel-7402 and SMMC-7721 cells with CCT3 knocked down or overexpressed, as measured by an MTT-based proliferation assay (B), caspase 3/7 Glo luciferase reagent (C), and an anchorage-independent soft-agar colony formation assay (D-E), respectively. Scale bar, 500 μm

(F) CCT3 boosted tumour growth *in vivo*, as evaluated in mouse xenografts models. n=5/group.

(G) The effects of CCT3 on phosphorylation of YAP, LATS and MST, and the co-targets of YAP and TFCP2 in xenografts generated by Bel-7402 cells. The results were visualized by WB.

(H) CCT3 had no effects on the interaction between YAP and TFCP2, as measured by co-IP in xenograft generated by Bel-7402 cells.

The data are analysed from 3 independent experiments (except Figure. S2F). **, *p* < 0.01 indicates statistical significance. The data were analysed by a one-way ANOVA test from 3 independent experiments.

**Supplementary Figure S3.**

**
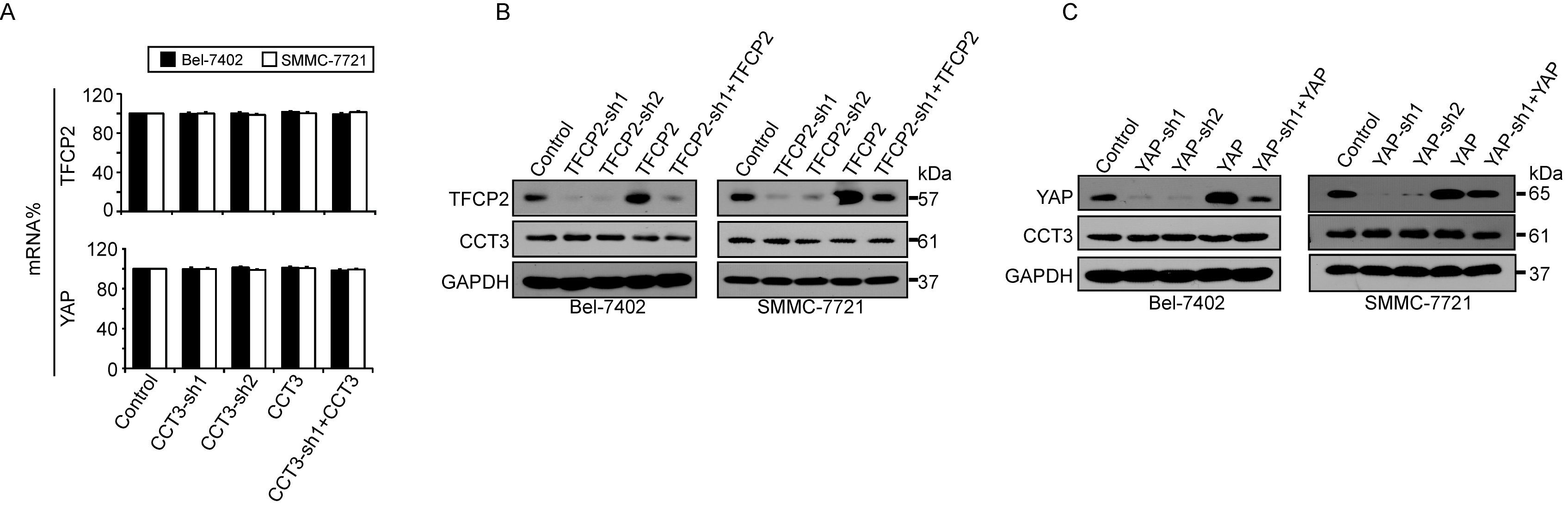
**

**Supplementary Figure S3. CCT3 did not regulate mRNA of YAP/TFCP2 and YAP/TFCP2 had no effects on CCT3**

(A) CCT3 played no roles on the mRNA expression of YAP/TFCP2 in both Bel-7402 and SMMC-7721 cells, as measured by qPCR.

(B) TFCP2 was unable to change the expression of CCT3, as measured by WB.

(C) YAP had no effects on the expression of CCT3, as measured by WB.

Images of WB and data from qPCR are ones from 3 independent experiments.

**Supplementary Figure S4.**

**
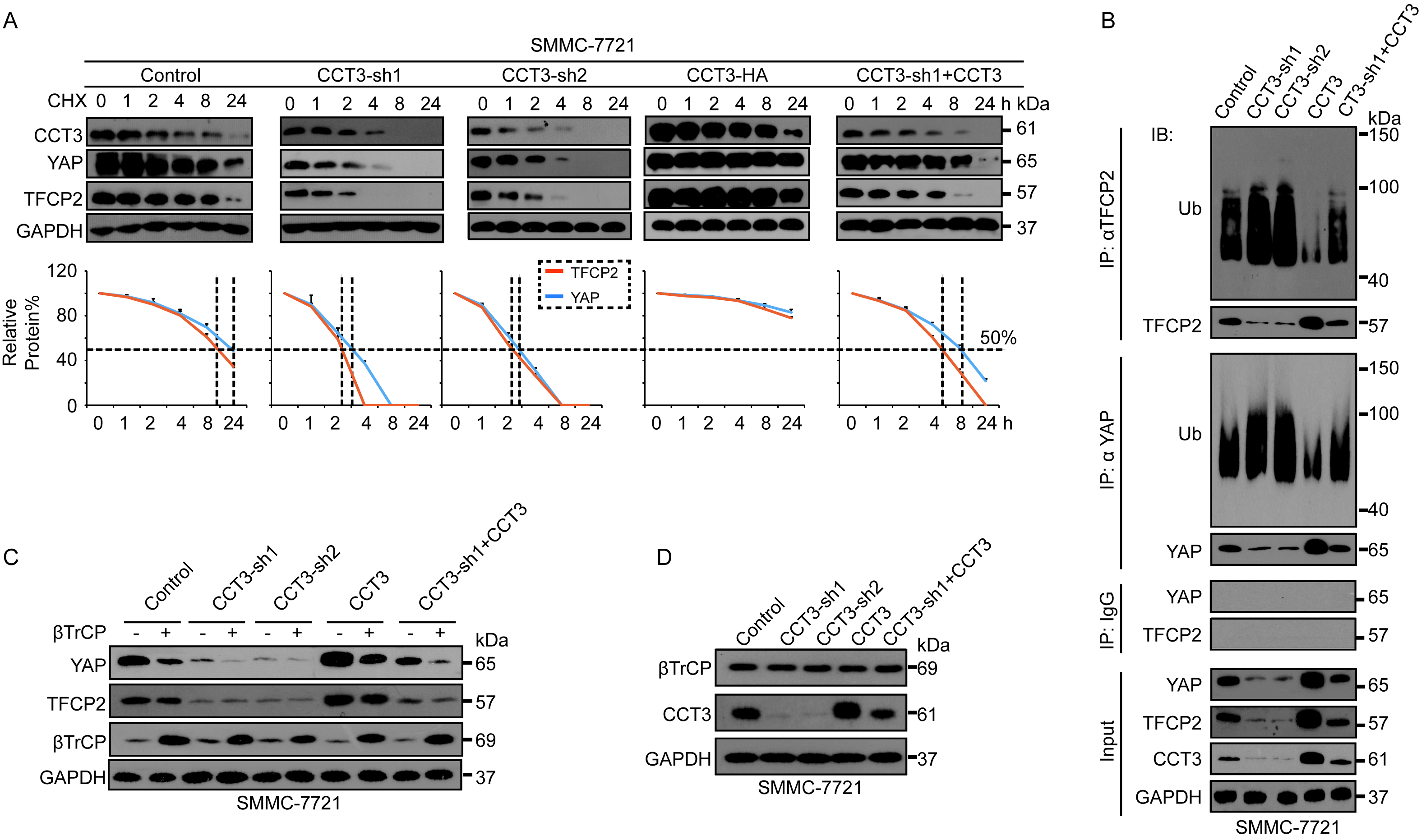
**

**Supplementary Figure S4. CCT3 affected protein stability and ubiquitination of YAP/TFCP2 in SMMC-7721 cells**

(A) CHX chase experiments on YAP and TFCP2 in control cells and SMMC-7721 cells with CCT3 knocked down in the presence or absence of CCT3 overexpression. The relative protein levels of YAP/TFCP2 were also normalized to that of GAPDH and plotted in the bottom. The “0h” points were arbitral set to 100%.

(B) Knockdown CCT3 increased ubiquitination of YAP/TFCP2 in SMMC-7721 cells.

(C) CCT3 could not affect the effects of βTrCP on YAP/TFCP2 in SMMC-7721 cells.

(D) CCT3 had no effects on the expression of βTrCP in SMMC-7721 cells.

Images of WB are representative ones from 3 independent experiments.

**Supplementary Figure S5.**

**
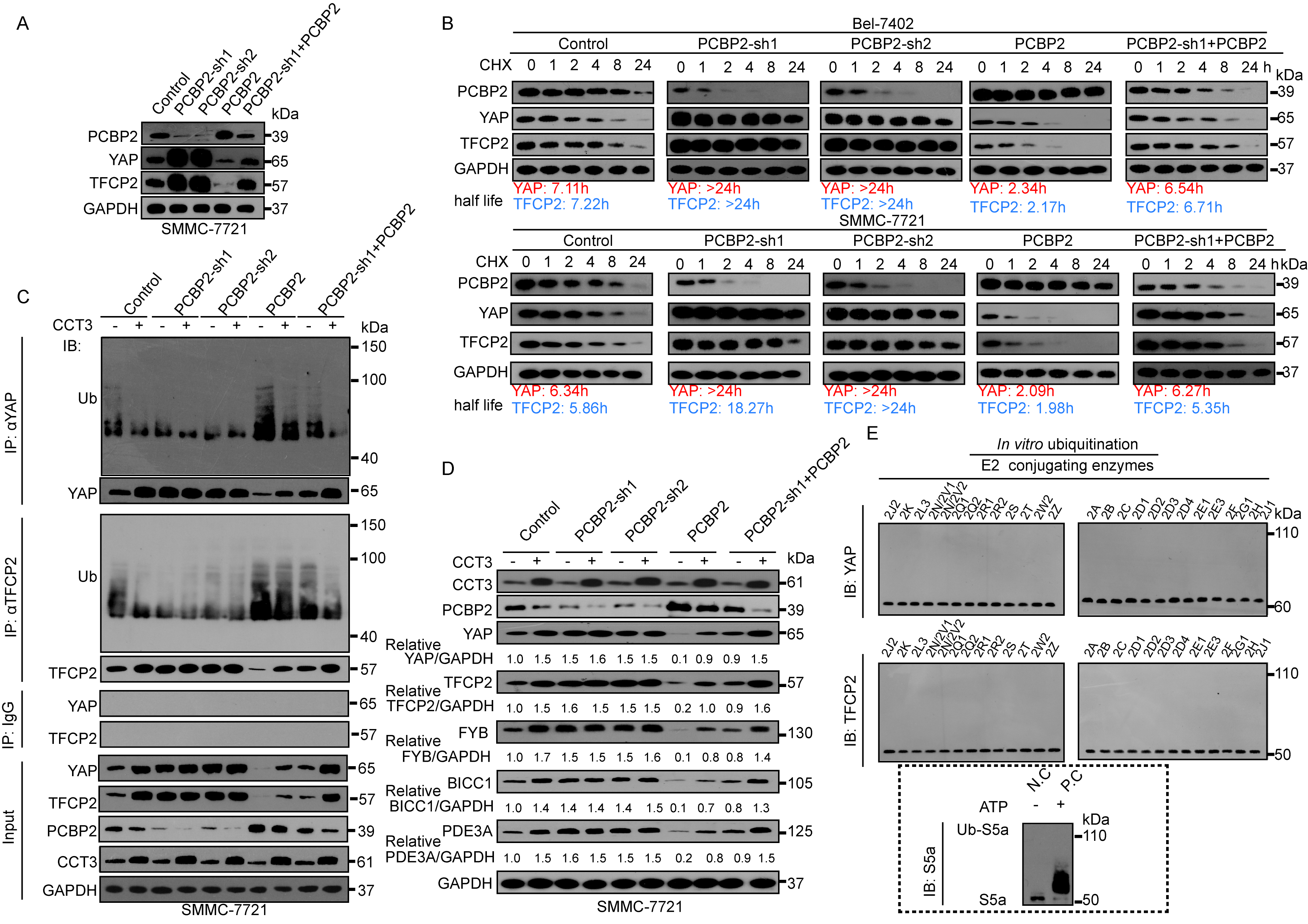
**

**Supplementary Figure S5. CCT3 regulated YAP/TFCP2 possibly via PCBP2**

(A) YAP and TFCP2 was negatively regulated by PCBP2 in SMMC-7721 cells.

(B) CHX chase of YAP and TFCP2 in control cells, Bel-7402 and SMMC-7721 cells with PCBP2 knocked down in the presence or absence of PCBP2 overexpression. The half-life of YAP and TFCP2 are shown at the bottom.

(C) CCT3 regulated ubiquitination of YAP and TFCP2 via PCBP2 in SMMC-7721 cells.

(D) CCT3 regulated YAP and TFCP2 and their targets via PCBP2 in control cells and SMMC-7721 cells with PCBP2 knocked down or overexpressed, as indicated.

(E) PCBP2 was not a ubiquitin E3 ligase, as measured by an *in vitro* ubiquitination assay. The indicated E2 was mixed with purified PCBP2 and YAP/TFCP2. The experiments were conducted by a kit from R&D system. The positive and negative controls were parallel performed by adding with or without ATP for the *in vitro* ubiquitination of S5a. The reagents for the control were provided within the kit. N.C., negative control and P.C., positive control.

All experiments are visualized by WB. Images of WB are representative ones from 3 independent experiments (except Figure S5E).
