# Supplementary material for: CCT3 acts upstream of YAP and TFCP2 as a potential target and tumour biomarker in liver cancer
Source: Cell Death Dis. 2019 Sep 9;10(9):644. doi: 10.1038/s41419-019-1894-5 (PMC6733791; doi:10.1038/s41419-019-1894-5)
Supplement: Supplementary file 2 — Supplementary table. S1 [file 41419_2019_1894_MOESM2_ESM.docx]

Supplementary Table. S1

| Protein name | Protein description |
| --- | --- |
| ABCD3 | ATP-binding cassette sub-family D member 3 |
| ACTA1 | Actin, alpha skeletal muscle |
| ACTA2 | Actin, aortic smooth muscle |
| ACTB | Actin, cytoplasmic 1 |
| ACTBL2 | Beta-actin-like protein 2 |
| ACTC1 | Actin, alpha cardiac muscle 1 |
| ACTN1 | Alpha-actinin-1 |
| AGPAT1 | 1-acyl-sn-glycerol-3-phosphate acyltransferase alpha |
| AHCY | Adenosylhomocysteinase |
| AIFM1 | Apoptosis-inducing factor 1, mitochondrial |
| ALB | Serum albumin |
| ALDOA | Fructose-bisphosphate aldolase A |
| AMOT | Angiomotin |
| AMOTL1 | Angiomotin-like protein 1 |
| ANXA5 | Annexin A5 |
| ARID3B | AT-rich interactive domain-containing protein 3B |
| ATAD3A | ATPase family AAA domain-containing protein 3A |
| ATAD3B | ATPase family AAA domain-containing protein 3B |
| ATP5A1 | ATP synthase subunit alpha, mitochondrial |
| ATP5B | ATP synthase subunit beta, mitochondrial |
| CALM1 | Calmodulin |
| CAPZA1 | F-actin-capping protein subunit alpha-1 |
| CCT2 | T-complex protein 1 subunit beta |
| CCT3 | T-complex protein 1 subunit gamma |
| CCT4 | T-complex protein 1 subunit delta |
| CCT6A | T-complex protein 1 subunit zeta |
| CCT8 | T-complex protein 1 subunit theta |
| CFL1 | Cofilin-1 |
| CKB | Creatine kinase B-type |
| CLTC | Clathrin heavy chain 1 |
| CLTCL1 | Clathrin heavy chain 2 |
| CORO1C | Coronin-1C |
| CPNE2 | Copine-2 |
| CPNE3 | Copine-3 |
| CTNNA1 | Catenin alpha-1 |
| DDX17 | Probable ATP-dependent RNA helicase DDX17 |
| DDX21 | Nucleolar RNA helicase 2 |
| DDX3X | ATP-dependent RNA helicase DDX3X |
| DDX5 | Probable ATP-dependent RNA helicase DDX5 |
| DHX9 | ATP-dependent RNA helicase A |
| DLD | Dihydrolipoyl dehydrogenase, mitochondrial |
| DPM1 | Dolichol-phosphate mannosyltransferase subunit 1 |
| DSG2 | Desmoglein-2 |
| DSP | Desmoplakin |
| DYNC1H1 | Cytoplasmic dynein 1 heavy chain 1 |
| EEF1A1 | Elongation factor 1-alpha 1 |
| EEF1A2 | Elongation factor 1-alpha 2 |
| EEF1B2 | Elongation factor 1-beta |
| EEF1G | Elongation factor 1-gamma |
| EEF2 | Elongation factor 2 |
| EIF3A | Eukaryotic translation initiation factor 3 subunit A |
| EIF4A1 | Eukaryotic initiation factor 4A-I |
| ENO1 | Alpha-enolase |
| ENO2 | Gamma-enolase |
| ENO3 | Beta-enolase |
| EPB41L2 | Band 4.1-like protein 2 |
| FASN | Fatty acid synthase |
| FLNB | Filamin-B |
| FLOT1 | Flotillin-1 |
| FLOT2 | Flotillin-2 |
| GAPDH | Glyceraldehyde-3-phosphate dehydrogenase |
| GFAP | Glial fibrillary acidic protein |
| GLB1 | Beta-galactosidase |
| GLO1 | Lactoylglutathione lyase |
| GNA11 | Guanine nucleotide-binding protein subunit alpha-11 |
| GNA12 | Guanine nucleotide-binding protein subunit alpha-12 |
| GNA13 | Guanine nucleotide-binding protein subunit alpha-13 |
| GNA14 | Guanine nucleotide-binding protein subunit alpha-14 |
| GNAI1 | Guanine nucleotide-binding protein G(i) subunit alpha-1 |
| GNAI2 | Guanine nucleotide-binding protein G(i) subunit alpha-2 |
| GNAI3 | Guanine nucleotide-binding protein G(k) subunit alpha |
| GNAO1 | Guanine nucleotide-binding protein G(o) subunit alpha |
| GNAS | Guanine nucleotide-binding protein G(s) subunit alpha isoforms XLas |
| GNAZ | Guanine nucleotide-binding protein G(z) subunit alpha |
| GNB1 | Guanine nucleotide-binding protein G(I)/G(S)/G(T) subunit beta-1 |
| GNB2 | Guanine nucleotide-binding protein G(I)/G(S)/G(T) subunit beta-2 |
| GNB4 | Guanine nucleotide-binding protein subunit beta-4 |
| GNG12 | Guanine nucleotide-binding protein G(I)/G(S)/G(O) subunit gamma-12 |
| GPC4 | Glypican-4 |
| GPI | Glucose-6-phosphate isomerase |
| GSN | Gelsolin |
| GTF2I | General transcription factor II-I |
| HADHA | Trifunctional enzyme subunit alpha, mitochondrial |
| HADHB | Trifunctional enzyme subunit beta, mitochondrial |
| HIST1H2AA | Histone H2A type 1-A |
| HIST1H2AB | Histone H2A type 1-B/E |
| HIST1H2BB | Histone H2B type 1-B |
| HIST1H4A | Histone H4 |
| HNRNPA1L2 | Heterogeneous nuclear ribonucleoprotein A1-like 2 |
| HNRNPC | Heterogeneous nuclear ribonucleoproteins C1/C2 |
| HNRNPCL1 | Heterogeneous nuclear ribonucleoprotein C-like 1 |
| HNRNPD | Heterogeneous nuclear ribonucleoprotein D0 |
| HNRNPK | Heterogeneous nuclear ribonucleoprotein K |
| HNRNPM | Heterogeneous nuclear ribonucleoprotein M |
| HNRNPR | Heterogeneous nuclear ribonucleoprotein R |
| HNRNPU | Heterogeneous nuclear ribonucleoprotein U |
| HSP90AA1 | Heat shock protein HSP 90-alpha |
| HSP90AA4P | Putative heat shock protein HSP 90-alpha A4 |
| HSP90AB1 | Heat shock protein HSP 90-beta |
| HSP90AB3P | Putative heat shock protein HSP 90-beta-3 |
| HSP90B1 | Endoplasmin |
| HSPA1A | Heat shock 70 kDa protein 1A |
| HSPA1L | Heat shock 70 kDa protein 1-like |
| HSPA2 | Heat shock-related 70 kDa protein 2 |
| HSPA4 | Heat shock 70 kDa protein 4 |
| HSPA5 | 78 kDa glucose-regulated protein |
| HSPA6 | Heat shock 70 kDa protein 6 |
| HSPA7 | Putative heat shock 70 kDa protein 7 |
| HSPA8 | Heat shock cognate 71 kDa protein |
| HSPA9 | Stress-70 protein, mitochondrial |
| HSPD1 | 60 kDa heat shock protein, mitochondrial |
| HSPH1 | Heat shock protein 105 kDa |
| ILF2 | Interleukin enhancer-binding factor 2 |
| IMPDH2 | Inosine-5~-monophosphate dehydrogenase 2 |
| INA | Alpha-internexin |
| IPO7 | Importin-7 |
| IRS4 | Insulin receptor substrate 4 |
| JUP | Junction plakoglobin |
| KHSRP | Far upstream element-binding protein 2 |
| KRT14 | Keratin, type I cytoskeletal 14 |
| KRT15 | Keratin, type I cytoskeletal 15 |
| KRT16 | Keratin, type I cytoskeletal 16 |
| KRT19 | Keratin, type I cytoskeletal 19 |
| KRT25 | Keratin, type I cytoskeletal 25 |
| KRT6A | Keratin, type II cytoskeletal 6A |
| KRT6B | Keratin, type II cytoskeletal 6B |
| KRT75 | Keratin, type II cytoskeletal 75 |
| KRT76 | Keratin, type II cytoskeletal 2 oral |
| KRT79 | Keratin, type II cytoskeletal 79 |
| LAMP2 | Lysosome-associated membrane glycoprotein 2 |
| LAMTOR1 | Ragulator complex protein LAMTOR1 |
| LAMTOR3 | Ragulator complex protein LAMTOR3 |
| LDHA | L-lactate dehydrogenase A chain |
| LDHB | L-lactate dehydrogenase B chain |
| LMNB1 | Lamin-B1 |
| LRRC59 | Leucine-rich repeat-containing protein 59 |
| LYN | Tyrosine-protein kinase Lyn |
| MARS | Methionine--tRNA ligase, cytoplasmic |
| MDH2 | Malate dehydrogenase, mitochondrial |
| MIF | Macrophage migration inhibitory factor |
| MPRIP | Myosin phosphatase Rho-interacting protein |
| MYH10 | Myosin-10 |
| MYH11 | Myosin-11 |
| MYH14 | Myosin-14 |
| MYH9 | Myosin-9 |
| MYL12A | Myosin regulatory light chain 12A |
| MYL6 | Myosin light polypeptide 6 |
| MYL6B | Myosin light chain 6B |
| MYL9 | Myosin regulatory light polypeptide 9 |
| MYO1A | Unconventional myosin-Ia |
| MYO1B | Unconventional myosin-Ib |
| MYO1C | Unconventional myosin-Ic |
| MYO1D | Unconventional myosin-Id |
| MYO3B | Myosin-IIIb |
| MYO6 | Unconventional myosin-VI |
| NAP1L1 | Nucleosome assembly protein 1-like 1 |
| NCL | Nucleolin |
| NPEPPS | Puromycin-sensitive aminopeptidase |
| OAT | Ornithine aminotransferase, mitochondrial |
| PABPC1 | Polyadenylate-binding protein 1 |
| PABPC3 | Polyadenylate-binding protein 3 |
| PARP1 | Poly [ADP-ribose] polymerase 1 |
| PCBP2 | Poly(rC)-binding protein 2 |
| PCMT1 | Protein-L-isoaspartate(D-aspartate) O-methyltransferase |
| PDHB | Pyruvate dehydrogenase E1 component subunit beta, mitochondrial |
| PGAM1 | Phosphoglycerate mutase 1 |
| PGK1 | Phosphoglycerate kinase 1 |
| PHB | Prohibitin |
| PHB2 | Prohibitin-2 |
| PHGDH | D-3-phosphoglycerate dehydrogenase |
| PKLR | Pyruvate kinase PKLR |
| PKM | Pyruvate kinase PKM |
| POTEE | POTE ankyrin domain family member E |
| POTEF | POTE ankyrin domain family member F |
| POTEI | POTE ankyrin domain family member I |
| POTEJ | POTE ankyrin domain family member J |
| POTEKP | Putative beta-actin-like protein 3 |
| PPIA | Peptidyl-prolyl cis-trans isomerase A |
| PRDX2 | Peroxiredoxin-2 |
| PRDX3 | Thioredoxin-dependent peroxide reductase, mitochondrial |
| PRDX6 | Peroxiredoxin-6 |
| PRKDC | DNA-dependent protein kinase catalytic subunit |
| PRPH | Peripherin |
| PSMA4 | Proteasome subunit alpha type-4 |
| RAB1A | Ras-related protein Rab-1A |
| RAI14 | Ankycorbin |
| RAN | GTP-binding nuclear protein Ran |
| RBM39 | RNA-binding protein 39 |
| RP2 | Protein XRP2 |
| RPA1 | Replication protein A 70 kDa DNA-binding subunit |
| RPL10A | 60S ribosomal protein L10a |
| RPL11 | 60S ribosomal protein L11 |
| RPL12 | 60S ribosomal protein L12 |
| RPL13 | 60S ribosomal protein L13 |
| RPL14 | 60S ribosomal protein L14 |
| RPL17 | 60S ribosomal protein L17 |
| RPL18 | 60S ribosomal protein L18 |
| RPL21 | 60S ribosomal protein L21 |
| RPL23 | 60S ribosomal protein L23 |
| RPL23A | 60S ribosomal protein L23a |
| RPL24 | 60S ribosomal protein L24 |
| RPL27 | 60S ribosomal protein L27 |
| RPL27A | 60S ribosomal protein L27a |
| RPL31 | 60S ribosomal protein L31 |
| RPL36 | 60S ribosomal protein L36 |
| RPL38 | 60S ribosomal protein L38 |
| RPL4 | 60S ribosomal protein L4 |
| RPL6 | 60S ribosomal protein L6 |
| RPL7 | 60S ribosomal protein L7 |
| RPL7A | 60S ribosomal protein L7a |
| RPLP0 | 60S acidic ribosomal protein P0 |
| RPLP0P6 | 60S acidic ribosomal protein P0-like |
| RPN1 | Dolichyl-diphosphooligosaccharide--protein glycosyltransferase subunit 1 |
| RPN2 | Dolichyl-diphosphooligosaccharide--protein glycosyltransferase subunit 2 |
| RPS10 | 40S ribosomal protein S10 |
| RPS13 | 40S ribosomal protein S13 |
| RPS14 | 40S ribosomal protein S14 |
| RPS15A | 40S ribosomal protein S15a |
| RPS16 | 40S ribosomal protein S16 |
| RPS17 | 40S ribosomal protein S17 |
| RPS18 | 40S ribosomal protein S18 |
| RPS19 | 40S ribosomal protein S19 |
| RPS2 | 40S ribosomal protein S2 |
| RPS23 | 40S ribosomal protein S23 |
| RPS24 | 40S ribosomal protein S24 |
| RPS25 | 40S ribosomal protein S25 |
| RPS26 | 40S ribosomal protein S26 |
| RPS28 | 40S ribosomal protein S28 |
| RPS3 | 40S ribosomal protein S3 |
| RPS3A | 40S ribosomal protein S3a |
| RPS4X | 40S ribosomal protein S4, X isoform |
| RPS5 | 40S ribosomal protein S5 |
| RPS7 | 40S ribosomal protein S7 |
| RPS8 | 40S ribosomal protein S8 |
| RPS9 | 40S ribosomal protein S9 |
| RPSA | 40S ribosomal protein SA |
| RUVBL1 | RuvB-like 1 |
| SLC1A5 | Neutral amino acid transporter B(0) |
| SLC25A31 | ADP/ATP translocase 4 |
| SLC25A4 | ADP/ATP translocase 1 |
| SLC25A5 | ADP/ATP translocase 2 |
| SLC25A6 | ADP/ATP translocase 3 |
| SPTAN1 | Spectrin alpha chain, non-erythrocytic 1 |
| SPTBN1 | Spectrin beta chain, non-erythrocytic 1 |
| SPTBN2 | Spectrin beta chain, non-erythrocytic 2 |
| STIP1 | Stress-induced-phosphoprotein 1 |
| SYNCRIP | Heterogeneous nuclear ribonucleoprotein Q |
| TARS | Threonine--tRNA ligase, cytoplasmic |
| TBB8L | Tubulin beta-8 chain-like protein LOC260334 |
| TCP1 | T-complex protein 1 subunit alpha |
| TFCP2 | Alpha-globin transcription factor CP2 |
| TKT | Transketolase |
| TMEM109 | Transmembrane protein 109 |
| TMOD3 | Tropomodulin-3 |
| TP53 | Cellular tumor antigen p53 |
| TPI1 | Triosephosphate isomerase |
| TPM1 | Tropomyosin alpha-1 chain |
| TPM2 | Tropomyosin beta chain |
| TRAP1 | Heat shock protein 75 kDa, mitochondrial |
| TUBA1A | Tubulin alpha-1A chain |
| TUBA1B | Tubulin alpha-1B chain |
| TUBA1C | Tubulin alpha-1C chain |
| TUBA3C | Tubulin alpha-3C/D chain |
| TUBA3E | Tubulin alpha-3E chain |
| TUBA4A | Tubulin alpha-4A chain |
| TUBA4B | Putative tubulin-like protein alpha-4B |
| TUBA8 | Tubulin alpha-8 chain |
| TUBAL3 | Tubulin alpha chain-like 3 |
| TUBB | Tubulin beta chain |
| TUBB1 | Tubulin beta-1 chain |
| TUBB2A | Tubulin beta-2A chain |
| TUBB3 | Tubulin beta-3 chain |
| TUBB4A | Tubulin beta-4A chain |
| TUBB4B | Tubulin beta-4B chain |
| TUBB6 | Tubulin beta-6 chain |
| TUBB8 | Tubulin beta-8 chain |
| TUFM | Elongation factor Tu, mitochondrial |
| TXN | Thioredoxin |
| UBA1 | Ubiquitin-like modifier-activating enzyme 1 |
| VCP | Transitional endoplasmic reticulum ATPase |
| VIM | Vimentin |
| WDR6 | WD repeat-containing protein 6 |
| XRCC6 | X-ray repair cross-complementing protein 6 |
| YAP1 | Transcriptional coactivator YAP1 |
| YBX1 | Nuclease-sensitive element-binding protein 1 |
| YES1 | Tyrosine-protein kinase Yes |
| YWHAB | 14-3-3 protein beta/alpha |
| YWHAQ | 14-3-3 protein theta |

**Supplementary table. S1.** The 228 candidates that co-interact with YAP and TFCP2 predicated by the mass spectrometry
